# Site‐Specific Iron Substitution in STA‐28, a Large Pore Aluminophosphate Zeotype Prepared by Using 1,10‐Phenanthrolines as Framework‐Bound Templates

**DOI:** 10.1002/anie.202005558

**Published:** 2020-06-08

**Authors:** Abigail E. Watts, Magdalena M. Lozinska, Alexandra M. Z. Slawin, Alvaro Mayoral, Daniel M. Dawson, Sharon E. Ashbrook, Bela E. Bode, A. Iulian Dugulan, Mervyn D. Shannon, Paul A. Cox, Alessandro Turrina, Paul A. Wright

**Affiliations:** ^1^ EaStCHEM School of Chemistry University of St Andrews Purdie Building North Haugh St Andrews, Fife KY16 9ST UK; ^2^ Instituto de Ciencia de Materiales de Aragon (ICMA) CSIC Universidad de Zaragoza Mariano Esquillor 50018 Zaragoza Spain; ^3^ Center for High-Resolution Electron Microscopy (*CħEM*) School of Physical Science and Technology ShanghaiTech University 393 Middle Huaxia Road Pudong Shanghai 201210 China; ^4^ Fundamental Aspects of Materials and Energy Group Delft University of Technology 2629 JB Delft The Netherlands; ^5^ Johnson Matthey Technology Centre Chilton P.O. Box 1, Belasis Avenue Billingham TS23 1LB UK; ^6^ School of Pharmacy and Biomedical Sciences University of Portsmouth St. Michael's Building, White Swan Road Portsmouth PO1 UK

**Keywords:** ADF STEM, aluminophosphate, framework-bound template, iron substitution, zeotype

## Abstract

An AlPO_4_ zeotype has been prepared using the aromatic diamine 1,10‐phenanthroline and some of its methylated analogues as templates. In each case the two template N atoms bind to a specific framework Al site to expand its coordination to the unusual octahedral AlO_4_N_2_ environment. Furthermore, using this framework‐bound template, Fe atoms can be included selectively at this site in the framework by direct synthesis, as confirmed by annular dark field scanning transmission electron microscopy and Rietveld refinement. Calcination removes the organic molecules to give large pore framework solids, with BET surface areas up to 540 m^2^ g^−1^ and two perpendicular sets of channels that intersect to give pore space connected by 12‐ring openings along all crystallographic directions.

## Graphical abstract

**The aromatic diamine** 1,10‐phenanthroline acts as a framework‐bound template for the large‐pore aluminophosphate zeotype STA‐28 and can be used to introduce Fe for Al at a single crystallographic site, as verified by Rietveld refinement and annular dark field scanning transmission electron microscopy. Removal of the template leaves a crystalline microporous solid with accessible iron cations.

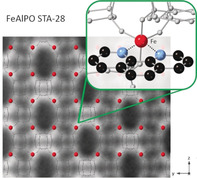

Microporous aluminophosphate‐based materials^1^ are of interest as adsorbents and catalysts,^2^, ^3^ so that there is an ongoing effort to prepare new framework types and compositions with novel properties.^4^ Aluminophosphates (AlPOs, Al:P=1:1) have zeolite‐like frameworks, where each Al is bonded to four phosphate tetrahedra. Substitutions of Al and P are possible: this can be either aliovalent (e.g. M^2+^ for Al, Si for P) or isovalent (Fe^3+^ for Al).^5^ Incorporation of metal cations into AlPO frameworks can introduce catalytic activity^6^ and there is interest in being able to control the location of substituting cations and so of catalytic sites. The related challenge of locating Al in zeolites is an ongoing research area: significant progress has been made in preparing materials that have differing Al distributions, as assessed indirectly from their catalytic properties,^7^ for example. Preferred Al positions have also been inferred from the scanning transmission electron microscopic (STEM) observation of Mo atoms thought to bind at framework O atoms adjacent to Al.^8^ However, direct imaging of dopant cations placed at a given site during synthesis has not yet been reported.

AlPOs are typically prepared hydrothermally in the presence of amines and alkylammonium cations as organic structure directing agents (OSDAs) or templates, which control their crystallisation.^4^ Linear and cyclic polyamines can be used as templates for aluminophosphates, either complexed or uncomplexed: the Cu^2+^ complex with cyclam gives SAPO STA‐7,^9^ for example. In such cases the metal complex remains in the zeotype cages after crystallisation, but there are no bonds to the framework. Upon calcination Cu^2+^ cations disperse into extra‐framework positions.

Here we describe the synthesis of an AlPO zeotype, STA‐28, using as the OSDA the aromatic diamine 1,10‐phenanthroline (1,10‐phen) and some of its methylated analogues (Scheme 1). 1,10‐Phen is known for its strong complexation properties, particularly in *tris*‐1,10‐phen complexes of Fe^2+^ and Fe^3+^.^10^ Remarkably, in the AlPO synthesis, the 1,10‐phen ends up bound to Al^3+^ cations at a particular crystallographic site in the zeotype framework, giving octahedral Al. It can be removed by calcination, leaving a microporous framework. Furthermore, having observed the framework Al complexation by 1,10‐phen in STA‐28, the use of diamine to introduce iron cations at a specific site was investigated via direct synthesis and the materials characterised by powder XRD and STEM.

**Scheme 1:**
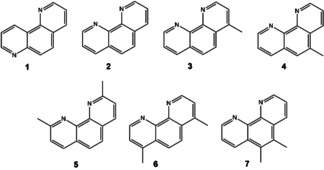
Phenanthrolines used in this study: **1**, 1,7‐phenanthroline (1,7‐phen); **2**, 1,10‐phen; **3**, 4‐methyl‐1,10‐phen; **4**, 5‐methyl‐1,10‐phen; **5**, 2,9‐dimethyl‐1,10‐phen; **6**, 4,7‐dimethyl‐1,10‐phen; **7**, 5,6‐dimethyl‐1,10‐phen.

As part of ongoing studies investigating OSDAs in AlPO crystallisation, 1,10‐phen and some of its analogues (Scheme 1) were added as potential templates.

Initial AlPO syntheses with 1,10‐phen (SI, sections S1 and S2) gave crystals suitable for single crystal XRD analysis and the structure was solved by direct methods and expanded using Fourier techniques.^11^ The new material, STA‐28 (St Andrews porous material‐28), crystallises in the body‐centred monoclinic space group *I*2/*a* (*a*=13.9291(8) Å, *b*=25.4248(13) Å, *c*=14.4085(8) Å, *β*=95.981(5)°). Details of the structure solution (*R*_int_=0.147, *R*1=0.077) and the crystal structure are given in the SI, sections S1 and S3 and the deposited cif file (Deposition Number 1989131 contains the supplementary crystallographic data for this paper. These data are provided free of charge by the joint Cambridge Crystallographic Data Centre and Fachinformationszentrum Karlsruhe Access Structures service www.ccdc.cam.ac.uk/structures). Rietveld analysis^12^ of powder X‐ray diffraction data (*R*_wp_=0.081), using the single crystal structure as a starting model and permitting restrained refinement of the framework and 1,10‐phen, confirmed that the product contains a single crystalline phase, unit cell formula Al_40_P_40_O_160_⋅8 C_12_N_2_H_8_⋅20 H_2_O (SI, section S4).

STA‐28 possesses an aluminophosphate framework with alternation of PO_4_ tetrahedra with either AlO_4_ tetrahedra or AlO_4_N_2_ octahedra, with the 1,10‐phen bound to framework Al (Figures 1, S4 and S5). There are 5 crystallographically‐distinct P atoms and 5 distinct Al atoms in the structure. P−O distances vary from 1.490 to 1.533 Å; tetrahedral Al−O distances from 1.706 to 1.739 Å, while in the octahedron, Al(5)−O distances are 1.809–1.871 Å, with both Al(5)−N distances 2.091(5) Å. The N atoms attached to the Al in the Al(5)O_4_N_2_ octahedron belong to a single molecule of 1,10‐phen, as illustrated in Figure 1. The average bond angle of the octahedron is 89.75°, with an esd of 6.8°, mainly from the ∡N‐Al‐N of 77.8° ^13^C MAS NMR spectrum is consistent with the presence of intact 1,10‐phen (Figure S8). Solid state ^27^Al MAS NMR spectroscopy of STA‐28 resolves four signals for 4‐coordinate Al and one for 6‐coordinate Al, while ^31^P MAS NMR gives two resonances with maxima at −26.6 and −30.9 ppm in a 2:3 integrated intensity ratio, indicating overlap of the signals from the five different P sites (see Figures S8 and S9).

**Figure 1:**
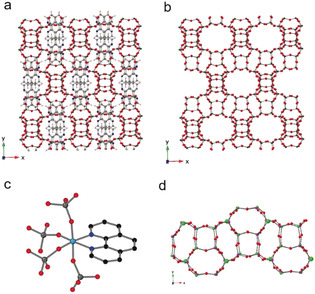
The structure of AlPO STA‐28. The framework structure viewed down the *z* axis, with (a) and without (b) 1,10‐phenanthroline shown; (c) the geometry at Al(5), showing the framework bound 1,10‐phenanthroline; (d) view of a structural “rod” comprising *d4r* and *lau* units.

In STA‐28, the 1,10‐phen molecules occupy space in straight 12R channels (each bounded by a ring of 12 Al or P atoms and 12 O atoms). These elliptical channels run along the *x* and *z* directions, as shown in Figures 1, S4 and S5. They have centres at heights of 1/4 and 3/4, and 0 and 1/2, respectively, in the unit cell. These channels intercept in such a way that there is also connectivity along the *y* direction via 12Rs linking the channels parallel to *x* and *z*, so that the pore space is three‐dimensionally connected. The 1,10‐phen molecules stack in the channels along the *z* axis 3.37 Å apart. Other examples of framework bound templates (Figure S10, SI section S6) include ECR‐40A,^13^ where three tris(2‐hydroxyethyl)methylammonium ions remain coordinated via O atoms to Al also bound to three phosphate O atoms and IST‐1, which has Al coordinated by via four Al‐O‐P linkages, a bridging hydroxyl and, unusually, a bound N (from methylamine).^14^

The AlPO_4_ framework comprises two topologically‐different types of secondary building unit, 4^6^ (*d4r*) and 4^2^6^4^ (*lau*) (in the as‐prepared structure there are two crystallographically distinct d4r units and three distinct *lau* units). These together make up similar “rods” arranged parallel to the *x* and *z* axes, sharing *lau* units with those “rods” above and below along the *y* axis, which are rotated by about 90° to their neighbours (Figure 1). The Al(5) sites open out onto the 12R channels (where they are coordinated by the phenanthroline molecules). The STA‐28 framework shares some similarities with the framework topologies SAO and ‐ITV (Figure S11). In the MgAPO STA‐1 (SAO),^15^ *lau*, *aww* and *sti* sub‐units connect to give 12R channels along *x* and *y* of the tetragonal unit cell that intersect to give 3D large pore connectivity, while the germanosilicate ITQ‐37 (‐ITV)^16^ framework also contains *lau* and *d4r* building units connected to give large channels.

To investigate whether other phenanthrolines could template STA‐28, the molecules of Scheme 1 were used. Neither 1,7‐phen nor 2,9‐dimethyl‐1,10‐phen gives STA‐28, because the N atoms are not in the correct configuration to coordinate to the framework in the former and because the methyl groups sterically prevent coordination in the latter. The 4‐ and 5‐methyl‐, and 4,7‐ and 5,6‐dimethyl‐1,10‐phen were successful, indicating there is space in the channels to accommodate both the phenanthroline group and the substituting methyl groups. Rietveld refinement (Figures S12, S13 and SI, section S7) showed that the unit cell expands as the framework adapts to take in substituted 1,10‐phen molecules.

In the AlPO_4_ composition, all “Al” sites are occupied by Al, but if 1,10‐phen could preferentially bind a cation different from Al then it would be possible to position it at a specific, pre‐determined site. Iron substitution into AlPOs is an attractive target, because when substituted into AlPO‐5, iron cations impart catalytic activity, for example in the Friedel–Crafts alkylation of benzene.^6^ Therefore, a series of STA‐28 samples was prepared in which attempts were made to substitute up to 30 % of the Al by Fe, using iron (II) acetate. Single phase, brick‐red, STA‐28(Fe) materials were formed with Fe/(Al+Fe) ratios up to 0.155 (by XRF) from the preparations with Fe/P=0.2 in the gel while above this iron content a second crystalline phase forms (Figure S14, SI sections S8). Additional experiments show that STA‐28(Fe) is also prepared using FeCl_3_ as the iron source (Figure S15). A range of spectroscopies was performed to determine the chemical environment and oxidation state of Fe in as‐prepared FeAlPO STA‐28 (SI, section S9). UV‐visible spectroscopy gave strong absorption, *λ*_max_=516 nm, resulting from coordination of iron with the 1,10‐phen. EPR spectra show a strong resonance (*g*=2.0) assigned as symmetric Fe^III^ by comparison with the literature.^17^ Mossbauer spectroscopy gave a resonance with an isomer shift of 0.41 mm s^−1^ and a quadrupole shift of 0.40 mm s^−1^ accounting for 98 % of the signal (Figure S18 and Table S12). Comparison with parameters of other Fe‐bearing solids^18^ suggests that the iron is present mainly as Fe^3+^ in an octahedral environment, suggesting oxidation of the Fe^II^ has taken place during synthesis and Fe^III^ is in the framework.

PXRD patterns of STA‐28(Fe) prepared with (Fe/P)_gel_=0.05–0.2 were analysed by Rietveld refinement, which indicated the unit cell size increased with Fe loading (SI section S10) and that the “Al(5)” site coordinated to 1,10‐phen was part‐occupied by Fe, whereas all other Al sites refined as Al. Constraining the total occupancy of site Al(5) to 1.0 suggested a content of Fe:Al of 0.73(2):0.27(2) in the STA‐28 with Fe/P=0.155 (R_wp_=0.037). Solid state ^27^Al NMR spectra showed both tetrahedral and octahedral Al species were present, supporting the partial occupancy of Fe in the Al(5) sites (SI), but the paramagnetism of Fe^III^ makes quantitative interpretation difficult (SI, section 9).

To visualise the structure of STA‐28 directly, ADF‐STEM imaging was performed using a spherical aberration corrected (C_s_‐corrected) high‐angle annular dark field FEI Titan 300 kV transmission electron microscope. The images of AlPO and FeAlPO analogues are shown in Figure 2, viewed down the 12R channels along [100]. Although these are of as‐prepared materials, only the heavy Al, P and Fe cations are visible.

**Figure 2:**
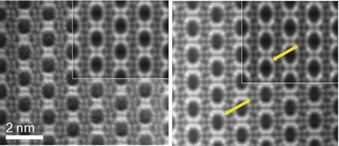
ADF‐STEM images along [100] of as‐prepared AlPO STA‐28 (left) and FeAlPO STA‐28 (right) processed as described in the SI, section S11, to give an averaged image. QSTEM simulations in which 0 % and 75 % of “Al(5)”, respectively, have been replaced by Fe are inset. Arrows indicate representative positions of Fe atoms in experimental and simulated images.

The images reveal the elliptical cross‐section of the 12R channels in the framework and the positions of framework cations. Most notably, the images from the FeAlPO show differences in their relative intensities from those of the AlPO. Comparison with images simulated using as models structures with and without Fe in the Al(5) site^19^ confirms that Fe is located preferentially in the Al(5) site, where it achieves coordination with the 1,10‐phen.

TGA of AlPO STA‐28(1,10‐phen) in flowing air shows that the template is removed above 600 °C. Calcination in air at 600 °C for 10 h resulted in some loss of crystallinity, but gave a material with microporosity of 0.15 cm^3^ g^−1^ (Figure S25). Solid state NMR indicated the Al was mainly tetrahedral (Figure S26). To minimise breakdown of the calcined STA‐28 structure associated with moisture uptake, a second route was adopted. STA‐28 was calcined in air, cooled and allowed to adsorb hexane. The solid retained crystallinity and N_2_ adsorption (adsorbed hexane removed) gave a pore volume of 0.21 cm^3^ g^−1^ (Figure S27).

To confirm the framework structure was retained upon calcination, a model was simulated for the empty structure starting from the single crystal structure. The 1,10‐phen was removed and the structure allowed to achieve an energy minimised configuration, without symmetry constraints, using the GULP program.^20^ This modelled “de‐templated” STA‐28 structure can best be described in the orthorhombic space group *Fddd* (SI, section S13) and all Al sites adopt tetrahedral geometry. This structure was used as a starting model for Rietveld refinement of the calcined material stabilised with hexane and then evacuated. A good fit was achieved to the PXRD pattern (*Fddd*, *a*=25.117(2) Å, *b*=20.248(3) Å, *c*=19.846(3) Å) indicating that the AlPO_4_ framework is retained upon calcination, although it has relaxed (Figures S29, S31). Notably, a pore volume of 0.26 cm^3^ g^−1^ was calculated for the structure, indicating some loss of porosity on calcination.

The calculated lattice energy of STA‐28 was compared with the lattice energies of other energy‐minimised AlPO_4_ polymorphs, expressed per AlPO_4_ unit. The latter were closely comparable to those reported previously for these materials.^21^ STA‐28 is less stable than other AlPO_4_ structures of similar framework density by ca. 4 kJ mol(AlPO_4_)^−1^ (Figure S28) and it is likely that this higher energy results from the distorted tetrahedral environment left around Al(5) of the original structure when 1,10‐phen is removed. The crystallisation of the STA‐28 framework is therefore facilitated by the 1,10‐phen. By contrast, 1,7‐phen, which is of similar shape to 1,10‐phen but with N atoms in positions that do not enable complexation of the Al cation, does not give STA‐28, suggesting that the extra stabilisation is required. Furthermore, if optimised as pure silica, the new framework type, although distorted, obeys the local interatomic distance criteria of Li et al,^22^ suggesting a silica polymorph is also feasible.

Finally, FeAlPO STA‐28 (prepared with 4‐methyl‐phen and with a refined Fe site occupancy of 0.45 in the as‐prepared form) was calcined to remove the template and stabilised by hexane loading as described previously. Activation gives a crystalline STA‐28 sample with a pore volume of 0.26 cm^3^ g^−1^, close to that predicted for the ideal structure. Furthermore, Rietveld refinement (Figure 3 and SI, section S14) indicates preferential Fe occupation in the same position (and with the same occupancy) as observed in the as‐prepared material. Using this as a starting point, the energy‐minimised structure of a site‐ordered Fe^3+^ cation in FeAl_4_P_5_O_20_ was calculated using DFT methods.^23^ The local environment of the Fe^3+^ cation in this simulated structure (Figure 3) has distorted tetrahedral geometry with its largest ∡OFeO angle of 146.1° opening out into the large channels (Table S18).

**Figure 3:**
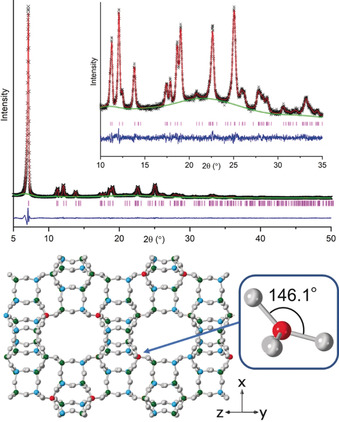
Above: Rietveld refinement plot of calcined, dehydrated FeAlPO STA‐28, R_wp_=0.038; below: measured FeAPO STA‐28 framework and, inset, DFT‐modelled geometry around the Fe^3+^.

In conclusion, 1,10‐phen and methylated derivatives act as framework‐bound templates for an AlPO_4_ in which they expand the coordination of a crystallographically‐distinct framework Al from tetrahedral AlO_4_ to octahedral AlO_4_N_2_. This behaviour can be exploited to direct Fe atoms into this site preferentially, as shown by STEM, Rietveld refinement and a range of spectroscopies. The template can be removed to give a microporous solid with pore space connected three dimensionally via 12R channels, while the Al or Fe introduced as complexed cations is subsequently left in a readily accessible location. This opens up the possibility of using framework‐bound templates to position catalytic metal cations in specific sites by direct synthesis.

## Conflict of interest

The authors declare no conflict of interest.

## Supporting information

As a service to our authors and readers, this journal provides supporting information supplied by the authors. Such materials are peer reviewed and may be re‐organized for online delivery, but are not copy‐edited or typeset. Technical support issues arising from supporting information (other than missing files) should be addressed to the authors.

Supplementary
