# Supplementary material for: Site‐Specific Iron Substitution in STA‐28, a Large Pore Aluminophosphate Zeotype Prepared by Using 1,10‐Phenanthrolines as Framework‐Bound Templates
Source: Angew Chem Int Ed Engl. 2020 Jun 8;59(35):15186–90. doi: 10.1002/anie.202005558 (PMC7496423; doi:10.1002/anie.202005558)
Supplement: Supplementary file 1 — Supplementary [file ANIE-59-15186-s001.pdf]

Angewandte Chemie
Eine Zeitschrift der Gesellschaft Deutscher Chemiker

Supporting Information

Site-Specific Iron Substitution in STA-28, a Large Pore Aluminophosphate Zeotype Prepared by Using 1,10-Phenanthrolines as Framework-Bound Templates

Abigail E. Watts, Magdalena M. Lozinska, Alexandra M. Z. Slawin, Alvaro Mayoral, Daniel M. Dawson, Sharon E. Ashbrook, Bela E. Bode, A. Iulian Dugulan, Mervyn D. Shannon, Paul A. Cox, Alessandro Turrina, and Paul A. Wright**

anie_202005558_sm_miscellaneous_information.pdf

Supporting Information

Contents

Experimental:

S1. Synthesis and characterisation; modelling

Results:

S2. Synthesis of AlPO STA-28

S3. Single crystal XRD of STA-28: Representation of the structure

S4. Rietveld analysis of AlPO STA-28 (1,10-phenanthroline)

S5. AlPO-STA-28(1,10-phen): Chemical and thermal analysis; Solid state NMR

S6. Structural comparison of STA-28

S7. Rietveld refinements of AlPOs prepared with other phenanthrolines

S8. Synthesis and analysis of FeAlPO STA-28

S9. Spectroscopy of FeAlPO STA-28 (Fe/P 0.20 in starting gel, 0.155 in product)

S10. Rietveld refinement on FeAlPO STA-28

S11. STEM on FeAlPO (and $AlPO_4$) STA-28

S12. Characterisation of calcined STA-28

S13. Energy minimised structures of AlPO and FeAlPO

S14. Rietveld refinement on calcined materials

References

Experimental:

S1. Synthesis and characterisation; modelling

The reagents used for the syntheses included phosphoric acid (85%, Alfa Aesar), aluminium hydroxide (Alfa Aesar), fumed silica (powder 0.007 µm, Aldrich), 1,7-phenanthroline, 1,10-phenanthroline, 4-methyl-1,10-phenanthroline, 5-methyl-1,10-phenanthroline, 2,9-dimethyl-1,10-phenathroline, 4,7-dimethyl-1,10-phenanthroline, 5,6-dimethyl-1,10-phenanthroline (all from Aldrich), iron (II) acetate (95%, Aldrich), iron(III) chloride (Aldrich) and tetrabutylammonium hydroxide solution (TBAOH, 55 wt. % in H_2O, Sachem). The gel compositions used in AlPO STA-28 preparations are given in Table S1. Tetrabutylammonium hydroxide was used as pH modifier, and chosen as a co-base because its shape and dimensions are not suitable to drive the formation of secondary phases.[S1]

Table S1. Gel compositions, conditions and products of AlPO syntheses. In the molar ratios, Al = $Al(OH)_3$, P = H_3PO_4, Si = SiO_2, R = phenanthroline

**Gel composition**<br>Al: P: Si: H_2O: R: TBAOH	**R**	**Temp. (°C)**	**Time (day)**	**Product**
1.0: 0.95: 0.05: 40: 0.1: 0.61	1,10-phenanthroline	180	4 – 7	STA-28
1.0: 0.95: 0.05: 40: 0.1: 0.61	1,7-phenanthroline	180	4 – 7	Dense phase
1.0: 0.95: 0.05: 40: 0.1: 0.61	2,9-dimethyl-1,10-phenanthroline	180	4 – 7	Dense phase
1.0: 0.95: 0.05: 40: 0.1: 0.61	4-methyl-1,10 phenanthroline	180	4 – 7	STA-28
1.0: 0.95: 0.05: 40: 0.1: 0.61	5-methyl-1,10-phenanthroline	180	4 – 7	STA-28
1.0: 0.95: 0.05: 40: 0.1: 0.61	4,7-dimethyl-1,10 phenanthroline	180	4 – 7	STA-28
1.0: 0.95: 0.05: 40: 0.1: 0.61	5,6-dimethyl-1,10-phenanthroline	180	4 – 7	STA-28

A typical gel was prepared by mixing H_3PO_4 (85%) with SiO_2 and $Al(OH)_3$ in water and stirred at room temperature. Then the selected phenanthroline template and metal acetate (if required) were added. TBAOH was used to reach a pH of 7. The final gels were stirred continuously at room temperature during the preparation procedure until homogeneous for at least 2 h, prior to transfer to a 30 mL Teflon-lined stainless-steel autoclave and heated at 180 °C for between 4 and 7 days. The resultant products were suspended in water and sonicated

to force separation of crystalline material from a suspension of amorphous solid, which was removed by decanting. The crystalline materials were dried at 80 °C for 12 h.

X-ray powder patterns of as-prepared materials in the 2θ range 5−50° (step size 0.01°, timestep 160 s, 0.04 rad Soller, 45 kV, 35 mA) were recorded on a PANalytical Empyrean automated diffractometer equipped with a X'Celerator detector (Bragg−Brentano geometry, Cu $K_{\alpha 1}$ X-radiation, λ = 1.54056 Å, via a primary monochromator). For structural analysis and refinement of dehydrated calcined materials, samples were loaded in 0.7 mm diameter quartz glass capillaries and dehydrated at 170 °C under a vacuum of 10^{-5} mbar for 10 h before being sealed. X-ray powder diffractograms were then collected in Debye-Scherrer mode over a 2θ range 5–70° (step size 0.1°, time step 80 s, 40 kV, 35 mA) on a Stoe STAD I/P diffractometer with a primary monochromator and PSD detector using Cu $K_{\alpha 1}$ X-ray radiation (λ = 1.54056 Å).

Single-crystal X-ray diffraction data for structure solution were recorded at -148 ºC using a Rigaku XtaLAB P200 diffractometer and Cu $K_{\alpha 1}$ (λ = 1.54056 Å) radiation. The data were processed using CrysAlisPro (Rigaku Oxford Diffraction) software[S2] and corrected for Lorentz and polarization effects. The structure was solved using direct methods, with refinement carried out using SHELXL Version 2017/1.[S3] All other calculations were performed using the CrystalStructure crystallographic software.[S4]

Solid-state NMR spectra were recorded on a Bruker Avance III spectrometer equipped with a 9.4 T wide-bore superconducting magnet (Larmor frequencies of 400.1, 162.0, 104.3, and 100.6 MHz, respectively for ^{1}H, ^{31}P, ^{27}Al and ^{13}C). Samples were packed into 4 mm zirconia rotors and rotated at the magic angle at a rate of 10 to 14 kHz. The ^{13}C NMR spectrum was recorded with cross polarisation from ^{1}H. A contact pulse (ramped for ^{1}H) of 1.5 ms was used and high-power ($\upsilon_1 \approx 100$ kHz) TPPM-15 decoupling of ^{1}H was applied during acquisition. Signal averaging was carried out for 4096 transients with a recycle interval of 3 s. ^{27}Al MAS NMR spectra were recorded with a short pulse (*ca*. 15-30° inherent flip angle) to provide approximately quantitative spectra. Signal averaging was carried out for 128 transients with recycle intervals of 1 s. The ^{27}Al multiple-quantum (MQ) MAS spectrum was recorded using an amplitude-modulated z-filtered pulse sequence with signal averaging for 24 transients with a recycle interval of 1 s for each of 180 t_1 increments of 35.71 μs. The spectrum was sheared and referenced according to the work of Pike *et al.*[S5] ^{31}P NMR spectra were recorded with signal averaging for 16 transients with a recycle interval of 60 s (as-prepared material) or 32

transients with a recycle interval of 5 s (calcined, dehydrated material). Chemical shifts are reported in ppm relative to $Si(CH_3)_4$, 0.1 M $Al(NO_3)_3$, and 85% H_3PO_4, using L-alanine ($\delta(CH_3)$ = 20.5 ppm), $Al(acac)_3$ (δ_{iso} = 0.0 ppm, C_Q = 3.08 MHz) and BPO_4 (δ = –29.6 ppm) as secondary solid references.

Scanning electron microscopy was performed on a JEOL JSM-5600 SEM fitted with an Oxford INCA Energy 200 EDX analyser. Thermogravimetric analysis of as-prepared samples was carried out on a Stanton Redcroft STA-780 in a dry air flow with a heating ramp of 5 ºC min^{-1} up to 700 ºC. Elemental compositions were determined on a PANalytical Axios WDXRF (wavelength-dispersive X-ray fluorescence) spectrometer with a 4 kW Rh tube. Elemental analysis was carried out by Elemental Analysis Service, London Metropolitan University, United Kingdom. Solid state UV-Vis absorption spectra were recorded on a JACSO V-650 UV-visible spectrophotometer with a photomultiplier tube detector. All spectra were obtained over the wavelength range 200–900 nm with a bandwidth of 5.0 nm and rate of 200 nm min^{-1}. Transmission ^{57}Fe Mössbauer spectra were collected at 300 and 4.2 K with a sinusoidal velocity spectrometer using a $^{57}Co(Rh)$ source. Velocity calibration was carried out using an α-Fe foil. The source and the absorbing samples were kept at the same temperature during the measurements. The Mössbauer spectra were fitted using the Mosswinn 4.0 program.[S6] The broad spectral contributions were fitted using a Blume-Tjon magnetic relaxation model.[S7] The EPR spectra were obtained using a Bruker EMX 10/12 spectrometer operating at ~9 GHz with 100 kHz modulation frequency. Selected samples were contained in 4 mm OD quartz tubes (Wilmad lab-glass). Measurements were performed in an ELEXSYS Super High Sensitivity Probehead (Bruker ER4122SHQE). The EPR spectra were recorded at 20 °C using 1 mW microwave power, a 6000 G field sweep centred at 3500 G with 1024 points resolution, a time constant and conversion time of 40.96 ms each, a modulation amplitude of 1 G and a microwave frequency of 9.839 GHz.

Calcination was performed in a tube furnace in flowing air at 600 °C (ramp 5 °C min^{-1}) for 10 h. In an optimised process the air was replaced with flowing N_2 during cooling and when the sample reached 50 °C hexane vapour was added to the N_2 flow and allowed to adsorb for 20 mins. This stabilised the calcined samples to subsequent exposure to moist lab air. To establish their porosity, samples were heated under vacuum and adsorption isotherms for N_2 at −196.15 °C were collected using a Micromeritics Tristar II 3020.

Scanning transmission electron microscopy (STEM) was performed in a XFEG FEI Titan transmission electron microscope operated at 300 kV. The column is fitted with a CEOS corrector, aligned prior to investigations using a gold standard assuring a potential spatial resolution of 0.8 Å. The electron dose was controlled via a monochromator, which allowed the displacement of the crossover position within the monochromator and therefore enabled increase or decrease of the number of electrons in continuous mode in STEM configuration. For the current experiments, the beam current was usually ≈ 3.5 pA or lower, resulting in an electron dose of around 2000 $e^-/Å^2$. Prior to observation, samples were crushed using a mortar and pestle and suspended in ethanol. A few drops of the suspension were put on coated holey carbon microgrids and allowed to dry. The inner collection angle of the detector for imaging was 50 mrad.

STEM Modelling

Simulation of (Annular Dark-Field) ADF-STEM images was performed using QSTEM, a computer program written by Christoph Koch.[S8] It is freely available from his website at Humboldt University, Berlin. Parameters matching those for the XFEG FEI Titan were used. A convergence angle of 17.5 mrad and collection angle 50-200 mrad were used for the Fe-containing sample. The pixel step size chosen was 0.36 Å and the slice thickness 1.36 Å. A source size of 1.7 Å gave a similar atom image size to that in the experiment. The images were oversampled 5-fold and Poisson noise added corresponding to an electron dose 4 times the experimental one.

All experimental images have been ABSF (Average Background Subtraction Filter) filtered[S9] to reduce the noise in these low dose images. Low dose is required to avoid beam damage. These filtered images contain information local to the unit cell. To get an averaged image a mask of small discs surrounding each diffraction spot in the FFT of the ABSF image has been applied and the inverse FFT calculated. Generally the processing is applied to large images (up to 4k by 4k) and small regions extracted for presentation.

Crystallographic analysis

Single-crystal X-ray Diffraction

A colourless prismatic crystal having approximate dimensions of 0.03 × 0.03 × 0.03 mm was mounted in a loop. Cell constants and an orientation matrix for data collection corresponded to an *I*-centered monoclinic cell with dimensions: *a* = 13.9291(8) Å, *b* = 25.4248(13) Å, *c* = 14.4085(8) Å, β = 95.981(5) °, V = 5074.9(5) $Å^3$. For Z = 8 and F.W. = 791.08, the calculated density is 2.071 g/cm^3 (Table S2).

Table S2. Details of collection of SCXRD data for as-made STA-28 (1,10-phenanthroline).

**STA-28**	
OSDA used	1,10-phenanthroline
Chemical formula	$(C_{12}H_8N_2)\ Al_5P_5O_{20}$
Crystal system	monoclinic
Space group	*I*2/*a*
μ / cm^{-1}	61.186
a / Å	13.9291(8)
b / Å	25.4248(13)
c / Å	14.4085(8)
β / °	95.981(5)
V / $Å^3$	5074.9(5-148)
T / °C	-148
Z	8
ρ / g cm^{-3}	2.0701
λ / Å	1.54056
R(int)	0.1471
Unique reflection	5184
R_1	0.0770
$wR^2(F^2)$	0.2339

Based on the reflection conditions of *hkl (h+k+l*= 2*n* and *h*0*l: h* = 2*n)*, packing considerations, a statistical analysis of intensity distribution, and the successful solution and refinement of the structure, the space group was determined to be *I*2/*a* (#15). The data was collected at a temperature of –148 ± 1°C to a maximum 2θ value of 150.7°. There was a total of 30364 reflections, with 5184 of these being unique (R_{int} = 0.1471). The linear absorption coefficient, μ, for Cu $K_{\alpha1}$ radiation is 61.186 cm^{-1}. An empirical absorption correction was applied which resulted in transmission factors ranging from 0.602 to 0.835. The data was also corrected for Lorentz and polarization effects. The structure was solved by direct methods[S10] and expanded using Fourier techniques. The non-hydrogen atoms were refined anisotropically. Hydrogen atoms were refined using the riding model. The final cycle of full-matrix least-squares refinement[S3] on F[S11] was based on 5184 observed reflections and 397 variable

parameters and converged (largest parameter shift was 0.00 times its esd) with unweighted and weighted agreement factors of 0.0770 and 0.2339. The goodness of fit was 1.04 and unit weights were used. The maximum and minimum peaks on the final difference Fourier map corresponded to 0.83 and –0.62 $e^{-}/Å^3$, respectively. Neutral atom scattering factors were taken from International Tables for Crystallography (IT), Vol. C, Table 6.1.1.4.[S11] Anomalous dispersion effects were included in F_{calc}[S12]; the values for Δf' and Δf" were those of Creagh and McAuley.[S13]. The values for the mass attenuation coefficients are those of Creagh and Hubbell.[S14]

Powder X-ray Diffraction

Rietveld refinement of the structures of as-prepared STA-28 materials was carried out using the GSAS suite of programs and the EXPGUI graphical interface.[S15] The crystal structure of STA-28, synthesised using 1,10-phenanthroline and solved by SCXRD was used as the starting model for the refinements. The instrumental background was fitted automatically by using a Chebyschev function. The peak profiles were modelled using a Pseudo-Voigt function (type 2).[S15,S16] The framework Al-O, P-O, O-O(Al), and O-O(P) distances were soft constrained to 1.72 Å (σ = 0.020 Å), 1.50 Å (σ = 0.020 Å), 2.82 Å (σ = 0.005 Å), and 2.50 Å (σ = 0.005 Å), respectively. Typical bond constraints of the C-N and C-C distances were correspondingly 1.30 Å (σ = 0.001 Å) and 1.40 Å (σ = 0.001 Å). Constraints were also applied to C-C and C-N diagonals, *etc*. The Al-N distance was constrained to 2.10 Å (σ = 0.002 Å). An overall isotropic atomic displacement parameter was set to 0.002 $Å^2$ for the framework atoms and 0.001 $Å^2$ for all C and N atoms. The fractional occupancies were set to 1.00 for the phenanthroline SDA atoms. During the cycles of refinement the x, y, z coordinates of the SDAs atoms were allowed to refine together. Convergence was achieved by refining simultaneously all profile parameters, scale factor, lattice constants, 2θ zero-point, and atomic positional and atomic displacement parameters for the framework atoms and the occupancies of the SDAs atoms. For Rietveld refinement of PXRD data from as-made STA-28 using 4-methyl and 5-methyl-1,10-phenanthrolines, the same starting modelling obtained from SCXRD structure solution was used. Methyl groups were added to the phenanthroline molecules following the method depicted in Figure S1.

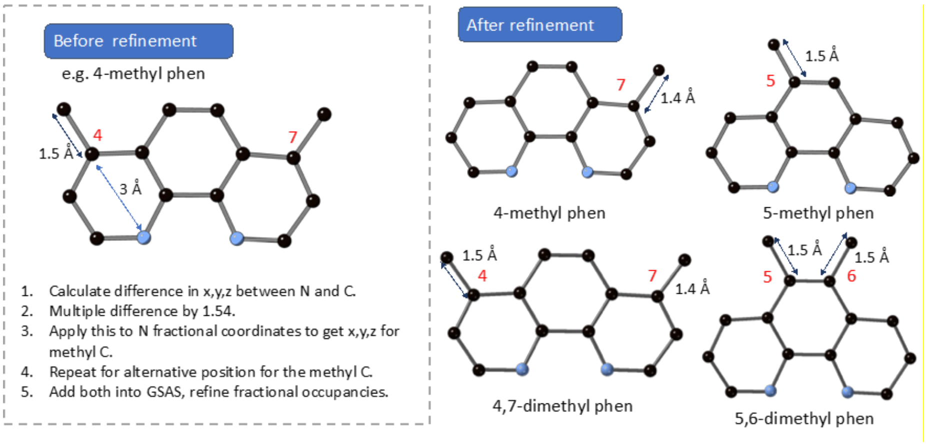

Figure S1. Method used in calculating the position of the methylated 1,10-phenanthroline molecules and the resulting positions of the methyl groups after refinement against PXRD data for the as-made materials.

Refinements were carried out following the procedure described above. For 4,7-dimethyl and 5,6-dimethyl 1,10-phenanthrolines, a similar method was used except the background was fitted using a Cosine-Fourier function and the peak profile modelled using a Pseudo-Voigt function (type 3).[S15] In the case of FeAlPO STA-28, the refinement process is the same as described earlier, with the added step of determining the Fe occupancy and location within the framework. The fractional occupancies of the Al atoms were refined one by one. The occupancies of the four tetrahedral Al sites stayed the same while the value for the octahedral Al site increased. This indicated that the Fe in the framework is located at the octahedral site. Thus, Fe was added into GSAS with the same fractional coordinates as the octahedral Al atom. The fractional occupancies of both this Al and the Fe atom were set to sum to one and subsequently refined. This gave the percentage of Fe present in the octahedral site within the STA-28 framework.

Rietveld refinement of dehydrated calcined $AlPO_4$ and FeAlPO STA-28 was performed in a similar way, with similar restraints on framework bond distances to begin with, which were gradually removed, and using as a starting model the energy-minimised fully tetrahedrally-connected structures in space group *Fddd* obtained using the program GULP[S17] (see below).

Computational Modelling

Energy minimisation of $AlPO_4$ and $FeAl_4P_5O_{20}$ STA-28 was performed using the program GULP.[S17] The simulation was carried out at constant pressure using the potentials derived by Sanders *et al.*[S18] Starting from the experimentally derived single crystal structure, the 1,10-phen OSDA was removed and energy minimisation was performed with no symmetry constraints applied. The best space group for the optimised structure was determined using the 'Find Symmetry' tool within the program Materials Studio[S19] (and found to be *Fddd*). The energy of $AlPO_4$ STA-28 was compared with those of other $AlPO_4$ frameworks.

The structure of FeAlPO STA-28 was also optimised via Density Functional Theory using the program CASTEP[S20] with the Generalized Gradient Approximation (GGA) and the Perdew-Burke-Emzerhof (PBE) exchange-correlation function. The structure obtained from Rietveld refinement for the calcined dehydrated FeAlPO STA-28 structure was used as the starting geometry. No symmetry constraints were applied during the calculation. The nearest space group for the optimised structure was *Fddd*.

Results:

S2. Synthesis of AlPO STA-28

*STA-28 (AlPO) 1,10-Phenanthroline I*n an optimised preparation, the STA-28 gel is prepared by mixing 1.16 g (11.86 mmol) of H_3PO_4 (85%, Alfa Aesar), 0.038 g (0.62 mmol) of fumed silica (0.007 µm powder, Aldrich) and 0.97 g (13 mmol) of $Al(OH)_3$ (Alfa Aesar) in 9 mL (500 mmol) of deionised water. 0.23 g (1.25 mmol) of 1,10-phenanthroline (Aldrich) was then added. The addition of 1.98 g (7.63 mmol) of TBAOH aqueous solution (55 wt% in H_2O, Sachem) is used to adjust the gel pH to 7. The gel is stirred for two hours at room temperature and loaded into a Teflon-lined 30 mL stainless-steel autoclave. Crystallisation is carried out at 180 °C for 4 - 7 days and the resultant products are suspended in water and sonicated to force separation of crystalline from amorphous solid, with the amorphous material removed by decanting. Then, the crystalline materials are dried under 80 °C for 12 hours. This gives approximately 1 g of an off-white solid. Only traces of Si (< 0.3 wt%) were found in the product.

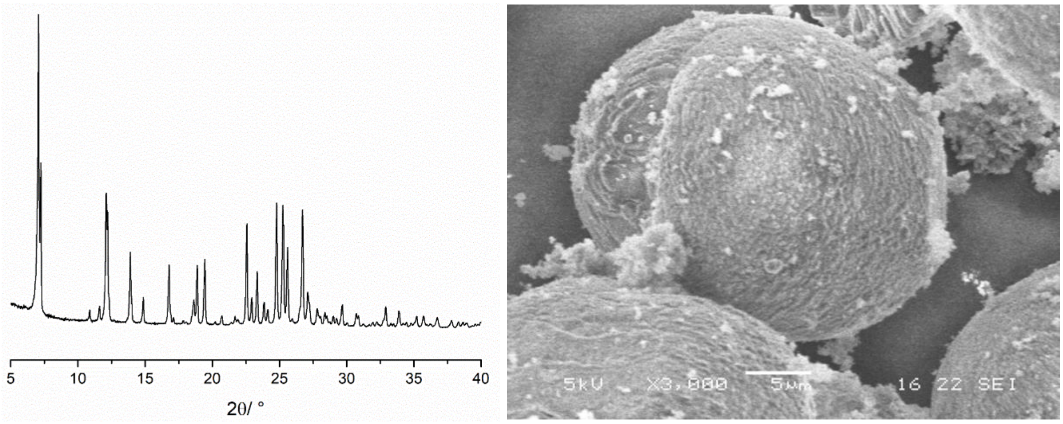

Figure S2. PXRD (left) and SEM image (right) of as-made STA-28 obtained with 0.05 Si/Al in the starting gel and 1,10-phenanthroline as structure directing agent.

STA-28 was prepared with a range of different 1,10-phenanthrolines, as described above. PXRD patterns of the successful syntheses of STA-28 are shown in Figure S3.

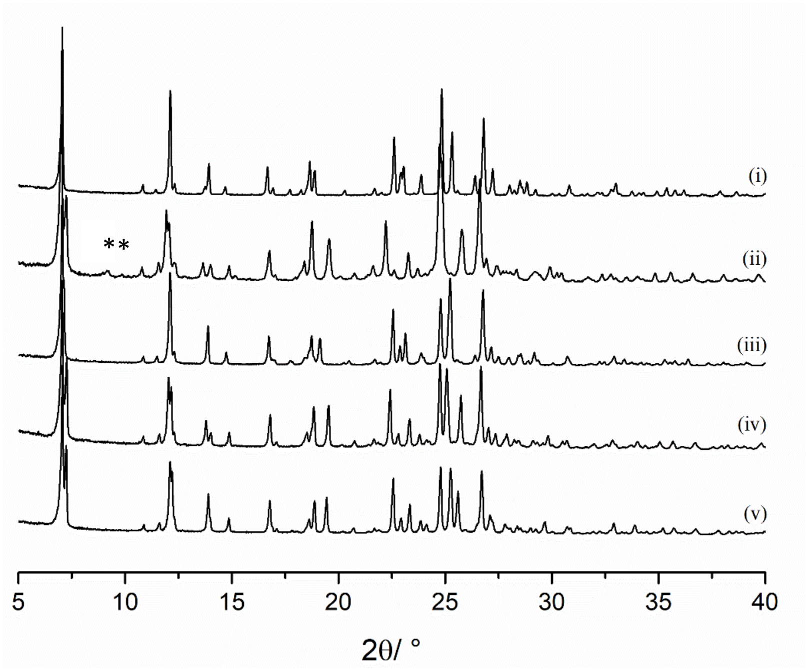

Figure S3. Powder X-ray diffraction patterns for as-made STA-28 prepared using a variety of structure directing agents. From upper to lower, the patterns represent STA-28 containing: (i) 5,6-dimethyl-1,10-phenanthroline; (ii) 4,7-dimethyl-1,10-phenanthroline (note the presence of minor amounts of an unidentified impurity, with reflections asterisked); (iii) 5-methyl-1,10-phenanthroline; (iv) 4-methyl-1,10-phenanthroline; and (v) 1,10-phenanthroline as templates.

S3. Single crystal XRD of STA-28: Representation of the structure

Table S3. Atomic coordinates and B_{eq} of non-H atoms from SXRD for as-made $AlPO_4$ STA-28.

Atom	x	y	z	B_{eq}
P1	0.35822(11)	0.28906(6)	0.62642(11)	1.95(3)
P2	0.12641(11)	0.16257(6)	0.59059(10)	1.88(3)
P3	0.03363(11)	0.37453(6)	0.69842(10)	1.98(3)
P4	0.13084(12)	0.58675(6)	0.59373(11)	2.21(3)
P5	0.35838(12)	0.45980(6)	0.62726(11)	2.20(3)
Al1	0.35312(13)	0.16581(7)	0.62093(12)	1.95(3)
Al2	0.13428(13)	0.28640(7)	0.58924(12)	1.93(3)
Al3	0.13405(14)	0.46186(7)	0.58535(13)	2.18(3)
Al4	0.35068(14)	0.58364(7)	0.62239(13)	2.22(3)
Al5	0.49294(12)	0.37488(8)	0.74517(11)	1.87(3)
O1	0.4195(3)	0.32092(17)	0.6963(3)	2.36(8)
O2	0.2515(4)	0.2963(2)	0.6392(4)	3.07(9)
O3	0.3748(4)	0.30480(19)	0.5274(3)	2.85(9)
O4	0.3838(4)	0.23111(18)	0.6414(4)	2.96(9)
O5	0.2319(4)	0.15426(19)	0.6278(4)	2.94(9)
O6	0.1014(4)	0.22102(17)	0.5982(3)	2.66(8)
O7	0.0605(3)	0.12901(17)	0.6414(3)	2.12(7)
O8	0.1149(4)	0.14727(19)	0.4876(3)	2.72(8)
O9	0.0583(3)	0.32419(17)	0.6480(3)	2.46(8)
O10	0.0656(4)	0.42376(17)	0.6503(3)	2.70(8)
O11	0.0805(3)	0.37335(17)	0.7988(3)	2.40(7)
O12	-0.0765(3)	0.37733(18)	0.6992(3)	2.33(7)
O13	0.1170(4)	0.52772(18)	0.6091(4)	2.97(9)
O14	0.2523(4)	0.44412(19)	0.6188(3)	3.00(9)
O15	0.2323(4)	0.60176(19)	0.6349(4)	3.01(9)
O16	0.1222(4)	0.59802(19)	0.4889(3)	2.97(9)
O17	0.0598(3)	0.61985(17)	0.6395(3)	2.33(7)
O18	0.3657(4)	0.51817(19)	0.6498(4)	3.08(9)
O19	0.4139(3)	0.42988(17)	0.7048(3)	2.28(8)
O20	0.3963(4)	0.4502(2)	0.5330(3)	2.97(9)
N1	0.6003(4)	0.3228(2)	0.8000(4)	2.15(9)
N10	0.5970(4)	0.4259(2)	0.8102(4)	2.24(9)
C1A	0.6828(5)	0.3458(2)	0.8414(4)	2.22(10)
C2	0.5970(5)	0.2703(3)	0.7998(5)	2.50(11)
C3	0.6744(6)	0.2396(3)	0.8393(5)	3.16(13)
C4	0.7566(5)	0.2621(3)	0.8781(5)	3.06(13)
C5	0.8464(6)	0.3474(3)	0.9185(5)	3.25(13)
C5A	0.7631(5)	0.3181(3)	0.8797(5)	2.58(11)
C6	0.8464(5)	0.4002(3)	0.9168(5)	3.09(13)
C6A	0.7626(5)	0.4296(3)	0.8818(5)	2.78(12)
C7	0.7572(6)	0.4851(3)	0.8821(6)	3.80(16)
C8	0.6736(7)	0.5088(3)	0.8491(5)	3.70(16)
C9	0.5925(6)	0.4781(3)	0.8150(5)	2.91(13)
C10A	0.6807(5)	0.4024(3)	0.8448(4)	2.28(11)

$B_{eq} = 8/3\ \pi^2(U_{11}(aa^*)^2 + U_{22}(bb^*)^2 + U_{33}(cc^*)^2 + 2U_{12}(aa^*bb^*)\cos\gamma + 2U_{13}(aa^*cc^*)\cos\beta + 2U_{23}(bb^*cc^*)\cos\alpha)$

Table S4. A summary of T-O bond distances within the STA-28 framework and C-C/C-N distances in 1,10-phenanthroline.

Atom		Distance (Å)	Atom		Distance (Å)
P1	O1	1.490(4)	P1	O2	1.527(5)
P1	O3	1.523(5)	P1	O4	1.526(5)
P2	O5	1.525(5)	P2	O6	1.533(5)
P2	O7	1.499(5)	P2	O8	1.526(5)
P3	Al4^{1}	2.871(2)	P3	O9	1.528(5)
P3	O10	1.520(5)	P3	O11	1.524(4)
P3	O12	1.537(5)	P4	O13	1.532(5)
P4	O15	1.524(5)	P4	O16	1.529(5)
P4	O17	1.503(5)	P5	O14	1.523(5)
P5	O18	1.520(5)	P5	O19	1.498(4)
P5	O20	1.527(5)	Al1	O4	1.732(5)
Al1	O5	1.727(5)	Al1	O8^{2}	1.736(5)
Al1	O11^{3}	1.721(5)	Al2	O2	1.733(5)
Al2	O3^{2}	1.736(5)	Al2	O6	1.733(5)
Al2	O9	1.717(5)	Al3	O10	1.706(5)
Al3	O13	1.731(5)	Al3	O14	1.727(5)
Al3	O20^{2}	1.739(5)	Al4	O12^{4}	1.734(5)
Al4	O15	1.739(6)	Al4	O16^{2}	1.725(5)
Al4	O18	1.719(5)	Al5	O1	1.809(5)
Al5	O7^{3}	1.868(5)	Al5	O17^{4}	1.871(5)
Al5	O19	1.836(5)	Al5	N1	2.090(5)
Al5	N10	2.092(6)	N1	C1A	1.369(8)
N1	C2	1.335(8)	N10	C9	1.330(8)
N10	C10A	1.358(8)	C1A	C5A	1.387(9)
C1A	C10A	1.439(9)	C2	C3	1.403(10)
C3	C4	1.348(10)	C4	C5A	1.427(10)
C5	C5A	1.441(10)	C5	C6	1.345(11)
C6	C6A	1.432(10)	C6A	C7	1.412(11)
C6A	C10A	1.393(10)	C7	C8	1.353(12)
C8	C9	1.418(11)			

Table S5. Bond angles in as-prepared STA-28

Atom			Angle (°)	Atom			Angle (°)
O1	P1	O2	110.4(3)	O1	P1	O3	111.0(3)
O1	P1	O4	108.7(3)	O2	P1	O3	109.0(3)
O2	P1	O4	108.4(3)	O3	P1	O4	109.3(3)
O5	P2	O6	109.1(3)	O5	P2	O7	111.5(3)
O5	P2	O8	107.8(3)	O6	P2	O7	111.2(3)
O6	P2	O8	108.4(3)	O7	P2	O8	108.8(3)
O9	P3	O10	112.5(3)	O9	P3	O11	109.8(2)
O9	P3	O12	108.4(3)	O10	P3	O11	109.4(3)
O10	P3	O12	107.8(3)	O11	P3	O12	108.8(3)
O13	P4	O15	108.3(3)	O13	P4	O16	109.2(3)
O13	P4	O17	112.7(3)	O15	P4	O16	108.2(3)
O15	P4	O17	108.4(3)	O16	P4	O17	109.9(3)
O14	P5	O18	108.4(3)	O14	P5	O19	110.4(3)
O14	P5	O20	108.0(3)	O18	P5	O19	108.5(3)
O18	P5	O20	109.0(3)	O19	P5	O20	112.5(3)
O4	Al1	O5	112.2(3)	O4	Al1	O8^{2}	109.4(3)
O4	Al1	O11^{3}	109.6(2)	O5	Al1	O8^{2}	110.5(2)
O5	Al1	O11^{3}	108.9(2)	O8^{2}	Al1	O11^{3}	106.2(2)
O2	Al2	O3^{2}	109.5(3)	O2	Al2	O6	110.7(2)
O2	Al2	O9	108.4(2)	O3^{2}	Al2	O6	109.7(2)

O3^{2}	Al2	O9	109.9(2)	O6	Al2	O9	108.6(2)
O10	Al3	O13	110.2(3)	O10	Al3	O14	105.7(2)
O10	Al3	O20^{2}	110.4(2)	O13	Al3	O14	110.1(2)
O13	Al3	O20^{2}	109.6(3)	O14	Al3	O20^{2}	110.8(3)
O12^{4}	Al4	O15	106.2(2)	O12^{4}	Al4	O16^{2}	107.6(2)
O12^{4}	Al4	O18	111.0(2)	O15	Al4	O16^{2}	110.0(3)
O15	Al4	O18	109.1(3)	O16^{2}	Al4	O18	112.7(3)
O1	Al5	O7^{3}	92.3(2)	O1	Al5	O17^{4}	92.7(2)
O1	Al5	O19	99.1(2)	O1	Al5	N1	91.2(2)
O1	Al5	N10	168.9(2)	O7^{3}	Al5	O17^{4}	173.5(2)
O7^{3}	Al5	O19	92.1(2)	O7^{3}	Al5	N1	88.3(2)
O7^{3}	Al5	N10	87.8(2)	O17^{4}	Al5	O19	91.2(2)
O17^{4}	Al5	N1	87.5(2)	O17^{4}	Al5	N10	86.5(2)
O19	Al5	N1	169.7(2)	O19	Al5	N10	92.0(2)
N1	Al5	N10	77.8(2)				
P1	O1	Al5	159.4(3)				
P1	O2	Al2	145.0(4)	P1	O3	Al2^{2}	146.4(3)
P1	O4	Al1	148.4(3)	P2	O5	Al1	150.2(4)
P2	O6	Al2	149.4(4)	P2	O7	Al5^{3}	141.0(3)
P2	O8	Al1^{2}	142.8(3)	P3	O9	Al2	152.1(3)
P3	O10	Al3	157.7(3)	P3	O11	Al1^{3}	142.8(3)
P3	O12	Al4^{1}	122.6(3)	P4	O13	Al3	153.8(4)
P5	O14	Al3	147.5(3)	P4	O15	Al4	138.1(3)
P4	O16	Al4^{2}	151.2(3)	P4	O17	Al5^{1}	143.5(3)
P5	O18	Al4	153.2(4)	P5	O19	Al5	150.3(3)
P5	O20	Al3^{2}	140.7(3)	Al5	N1	C1A	115.4(4)
Al5	N1	C2	127.5(4)	C1A	N1	C2	117.1(5)
Al5	N10	C9	127.4(5)	Al5	N10	C10A	114.8(4)
C9	N10	C10A	117.6(6)	N1	C1A	C5A	124.2(6)
N1	C1A	C10A	115.0(5)	C5A	C1A	C10A	120.8(6)
N1	C2	C3	122.0(6)	C2	C3	C4	121.1(7)
C3	C4	C5A	118.7(7)	C5A	C5	C6	120.6(7)
C1A	C5A	C4	116.9(6)	C1A	C5A	C5	118.4(6)
C4	C5A	C5	124.6(6)	C5	C6	C6A	121.8(7)
C6	C6A	C7	124.2(7)	C6	C6A	C10A	118.7(6)
C7	C6A	C10A	117.1(7)	C6A	C7	C8	119.2(8)
C7	C8	C9	120.2(7)	N10	C9	C8	121.7(7)
N10	C10A	C1A	116.5(6)	N10	C10A	C6A	124.1(6)
C1A	C10A	C6A	119.4(6)				

Illustration of STA-28 structure

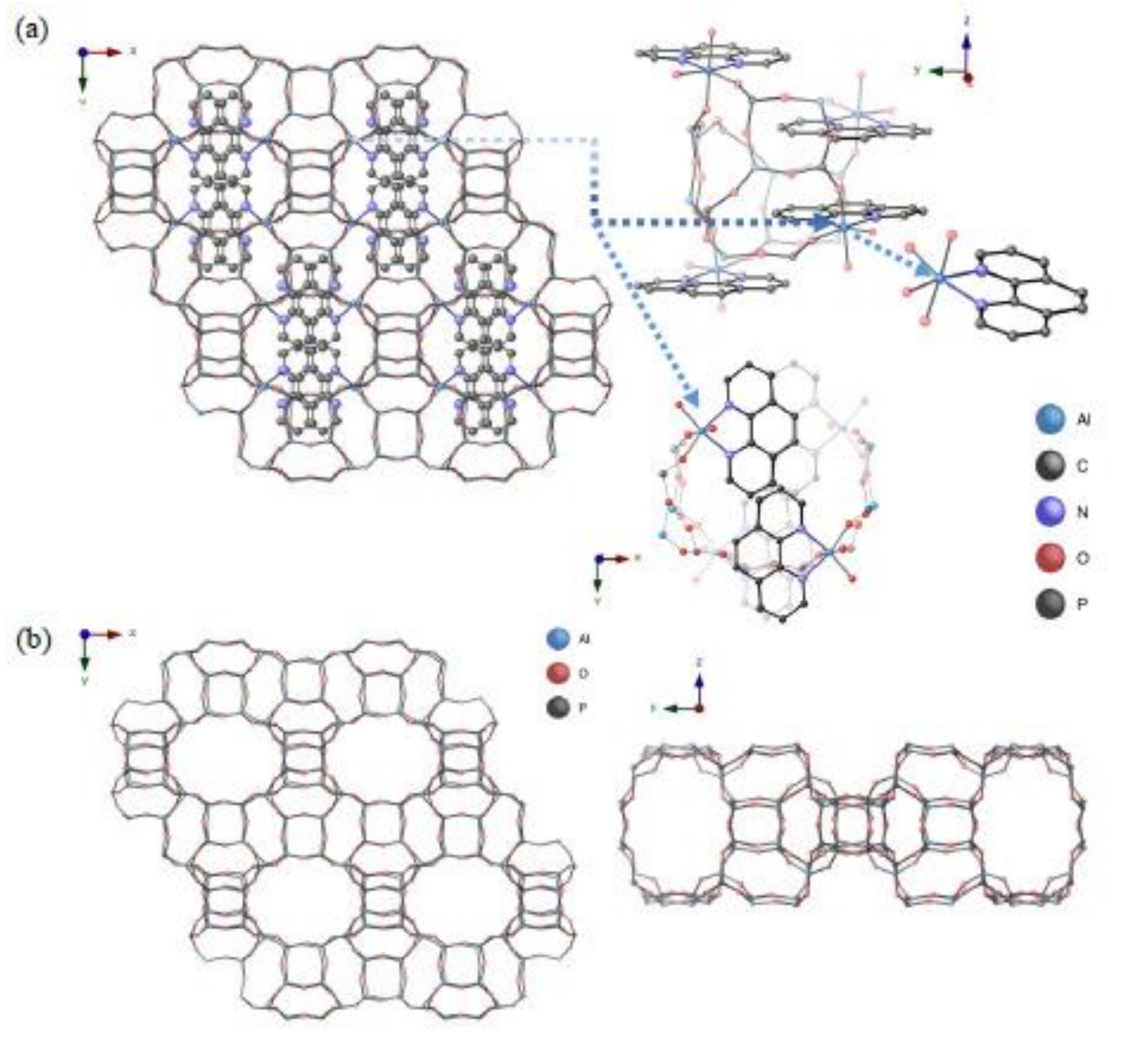

Figure S4. (a) As-made STA-28, showing the 1,10-phenanthroline bound to the framework, giving an octahedral Al site and (b) the framework of as-made STA-28 with template atoms omitted.

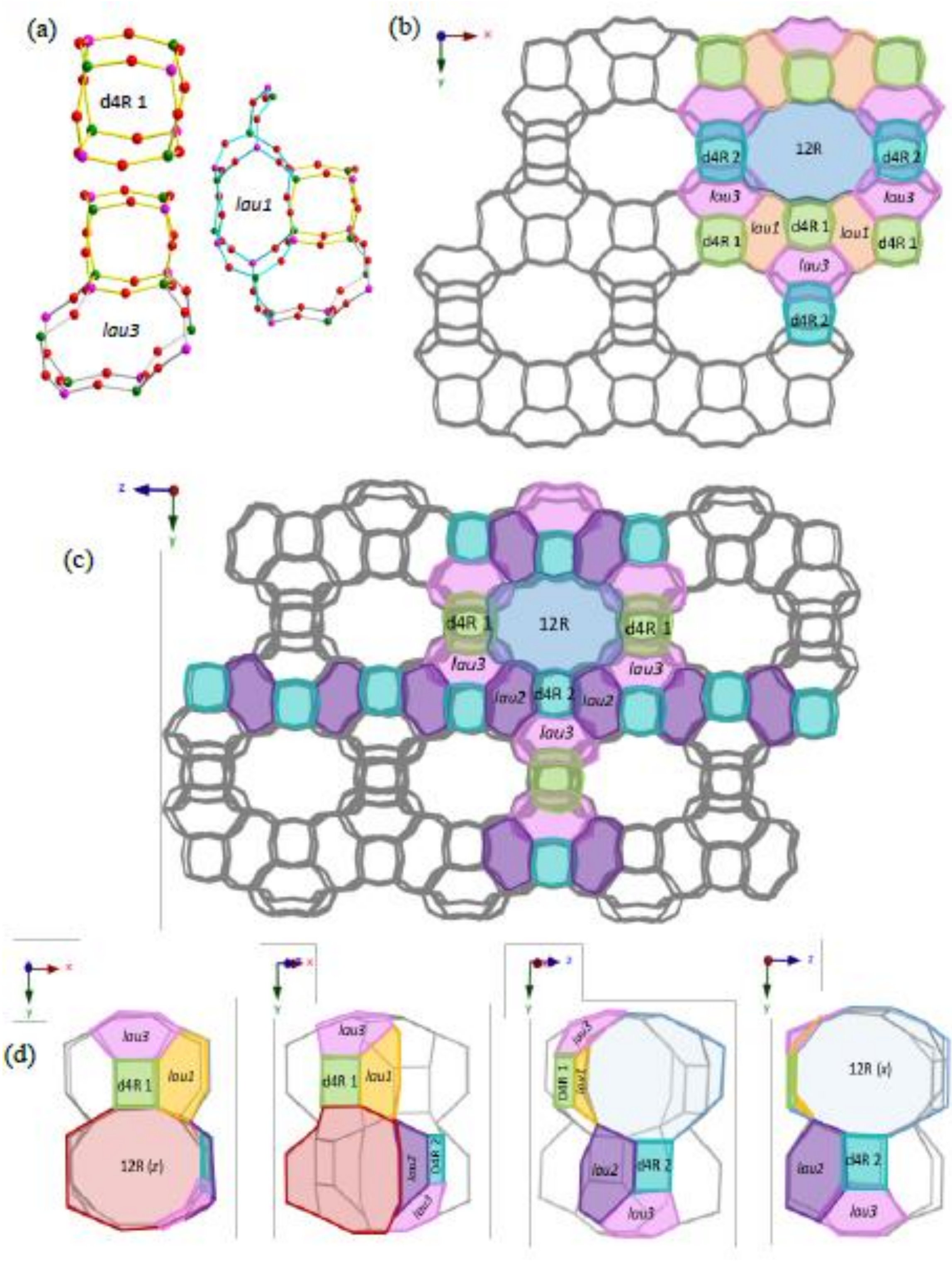

Figure S5. (a) The two types of SBUs found within the STA-28 framework, (b) and (c) show how the *d4r* and t-*lau* units are linked down the *z* and *x* axes, respectively and finally, (d) depicts how a 12R opening along *z* is connected to a 12R opening along *x*. This gives rise to the 3-dimensional connectivity observed for STA-28.

S4. Rietveld analysis of AlPO STA-28 (1,10-phenanthroline)

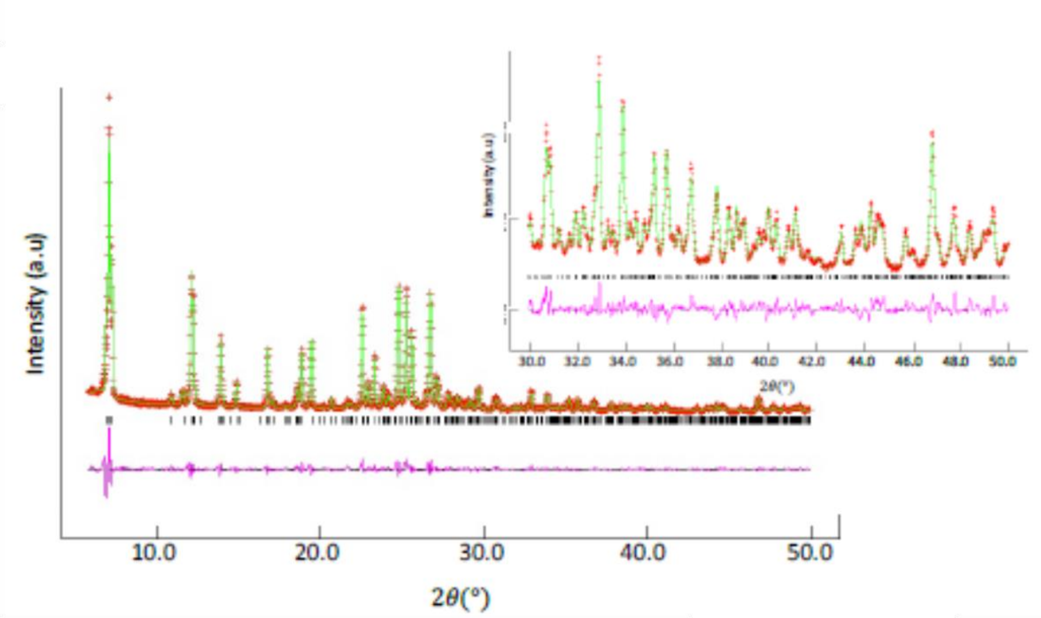

Figure S6. Restrained Rietveld refinement of as-made, hydrated STA-28 in space group *I* 2/*a*, using the structure determined by single crystal as a starting model (a = 13.9916(5) Å; b = 25.4790(9) Å; c = 14.4450(5) Å; β =95.9949(30)°, R_{wp} = 8.15%). [Red crosses = experimental data, green line = simulated data, black tick marks = predicted peak positions, magenta line = difference profile]

Table S6. Fractional atomic coordinates obtained from Rietveld refinement against PXRD data for as-made STA-28. (Uiso = 0.002 Å^2 for the framework atoms & 0.001 Å^2 for all C & N atoms).

Atom	Fractional Coordinates		
	x	y	z
**P1**	0.3592(15)	0.2903(9)	0.6247(16)
**P2**	0.1309(15)	0.1600(8)	0.5875(16)
**P3**	0.0302(14)	0.3727(10)	0.7022(15)
**P4**	0.1332(17)	0.5872(8)	0.5962(15)
**P5**	0.3606(16)	0.4573(9)	0.6324(16)
**Al1**	0.3539(16)	0.1647(9)	0.6189(18)
**Al2**	0.1362(19)	0.2843(10)	0.5899(18)
**Al3**	0.1321(19)	0.4585(10)	0.5883(17)
**Al4**	0.3521(17)	0.5855(8)	0.6274(17)
**Al5**	0.5008(11)	0.3775(8)	0.7546(14)
**O1**	0.4300(18)	0.3194(12)	0.6971(21)
**O2**	0.2526(19)	0.2992(15)	0.6304(26)
**O3**	0.3864(26)	0.2981(16)	0.5273(19)
**O4**	0.3765(26)	0.2311(9)	0.6415(31)
**O5**	0.2360(18)	0.1522(13)	0.6233(28)
**O6**	0.1167(30)	0.2181(9)	0.6093(27)
**O7**	0.0628(22)	0.1290(17)	0.6382(19)
**O8**	0.1064(25)	0.1490(15)	0.4857(18)
**O9**	0.0626(24)	0.3233(12)	0.6513(27)
**O10**	0.0651(25)	0.4198(13)	0.6541(26)
**O11**	0.0764(24)	0.3718(14)	0.8018(17)
**O12**	-0.0756(17)	0.3762(16)	0.7059(20)
**O13**	0.1086(28)	0.5270(9)	0.6123(27)
**O14**	0.2519(19)	0.4451(15)	0.6249(28)

**O15**	0.2329(17)	0.5992(13)	0.6387(28)
**O16**	0.1306(26)	0.5964(14)	0.4899(17)
**O17**	0.0578(21)	0.6163(17)	0.6446(20)
**O18**	0.3715(26)	0.5182(9)	0.6534(29)
**O19**	0.4189(17)	0.4339(12)	0.7177(20)
**O20**	0.3862(26)	0.4479(16)	0.5327(18)
**N1**	0.6065(4)	0.3290(4)	0.8062(4)
**N10**	0.6032(4)	0.4321(4)	0.8163(4)
**C1A**	0.6890(4)	0.3520(4)	0.8476(4)
**C2**	0.6032(4)	0.2766(4)	0.8060(4)
**C3**	0.6806(4)	0.2458(4)	0.8455(4)
**C4**	0.7628(4)	0.2683(4)	0.8843(4)
**C5**	0.8526(4)	0.3535(4)	0.9248(4)
**C5A**	0.7693(4)	0.3243(4)	0.8859(4)
**C6**	0.8525(4)	0.4065(4)	0.9231(4)
**C6A**	0.7688(4)	0.4358(4)	0.8880(4)
**C7**	0.7635(4)	0.4912(4)	0.8884(4)
**C8**	0.6798(4)	0.5150(4)	0.8552(4)
**C9**	0.5987(4)	0.4842(4)	0.8212(4)
**C10A**	0.6867(4)	0.4084(4)	0.8508(4)

P- O bond	Distance (Å)	Al- O bond	Distance (Å)
**P1- O1**	1.552(17)	**Al1- O4**	1.746(18)
**P1- O2**	1.519(18)	**Al1- O5**	1.689(18)
**P1-O3**	1.509(18)	**Al1-O8**	1.711(18)
**P1-O4**	1.543(18)	**Al1-O11**	1.700(19)
**P2-O5**	1.519(18)	**Al2-O2**	1.714(18)
**P2-O6**	1.532(18)	**Al2-O3**	1.725(18)
**P2-O7**	1.489(19)	**Al2-O6**	1.736(18)
**P2-O8**	1.501(18)	**Al2-O9**	1.739(18)
**P3-O9**	1.551(18)	**Al3-O10**	1.715(18)
**P3-O10**	1.493(18)	**Al3-O13**	1.818(18)
**P3-O11**	1.515(18)	**Al3-O14**	1.737(18)
**P3-O12**	1.489(18)	**Al3-O20**	1.760(18)
**P4-O13**	1.595(18)	**Al4-O12**	1.739(19)
**P4-O15**	1.496(18)	**Al4-O15**	1.729(18)
**P4-O16**	1.549(18)	**Al4-O16**	1.759(18)
**P4-O17**	1.518(19)	**Al4-O18**	1.769(18)
**P5-O14**	1.546(18)	**Al5-O1**	1.922(18)
**P5-O18**	1.585(18)	**Al5-O7**	1.871(17)
**P5-O19**	1.526(18)	**Al5-O17**	1.859(18)
**P5-O20**	1.538(18)	**Al5-O19**	1.880(18)

S5. AlPO-STA-28(1,10-phen): Chemical and Thermal Analysis; Solid state NMR

Chemical Analysis XRF gave Al/P = 1.03, with negligible Si content. Deviation from 1.00 assumed to be due to the presence of minor mounts of amorphous impurities (seen for example in the NMR). Calculation of the water content from the TGA weight loss (Figure S7) below 250 °C gives the idealised unit cell composition $Al_{40}P_{40}O_{160}$.8(1,10-phen).20H_2O. CHN analysis (wt%) gives: (calculated) C 17.2, H 1.5, N 3.3 vs. (observed) C 16.0, H 1.4, N 3.1.

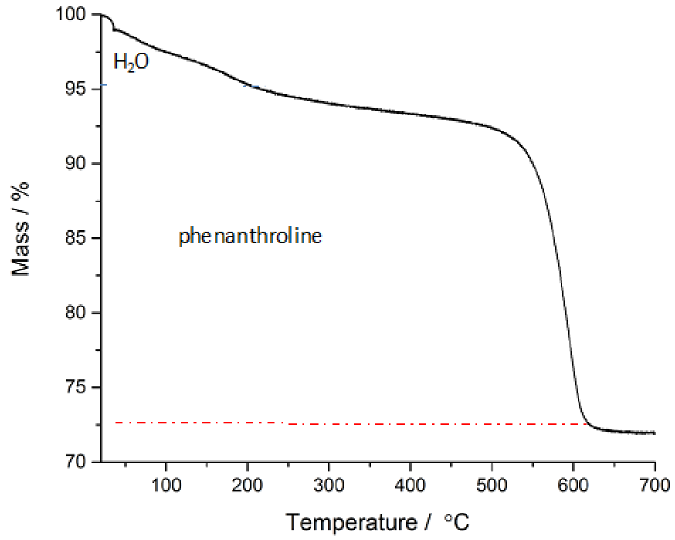

Figure S7. TGA analysis of STA-28(1,10-phen).

Solid State NMR

Figure S8 shows the solid-state ^{13}C, ^{27}Al and ^{31}P NMR spectra of STA-28(1,10-phen). The ^{13}C NMR spectrum (Figure S8(a)) is consistent with 1,10-phen bound to Al and is assigned as shown. The ^{27}Al MAS NMR spectrum (Figure S8(b)) shows a complex signal for tetrahedral Al, a sharp signal for octahedral Al, and a broad signal corresponding to pentacoordinate Al in an amorphous impurity. The ^{27}Al MQMAS spectrum shown in Figure S9 allows resolution of four distinct tetrahedral Al sites with isotropic chemical shifts of 47, 45, 41 and 40 ppm, and quadrupolar products ($P_Q = C_Q (1 + h_Q^2/3)^{1/2}$) of 1.9, 1.4, 1.6 and 2.3 MHz, respectively. The octahedral site has d_{iso} = –4.9 ppm and P_Q = 2.0 MHz.

The ^{31}P MAS NMR spectrum of as-prepared STA-28(1,10-phen), shown in Figure S8(c) contains two signals from the AlPO framework at –26.6 and –30.9 ppm in an approximately 2 : 3 integrated intensity ratio, consistent with the presence of five P sites. While assignment using DFT calculations was not possible (as the 20 H_2O per unit cell were not located in the crystal structure), the recently developed DISCO program[S21] can be used to provide an approximate prediction of ^{31}P δ_{iso}, based on the mean P-O-Al bond angles and P-O bond

lengths. The predicted chemical shifts for STA-28 are shown as red points in Figure S8(c), and it can be seen that three P signals are predicted to be at higher shift with two at lower shift, which is the opposite of the experimental result. Therefore, assignment using DISCO alone is ambiguous in this case.

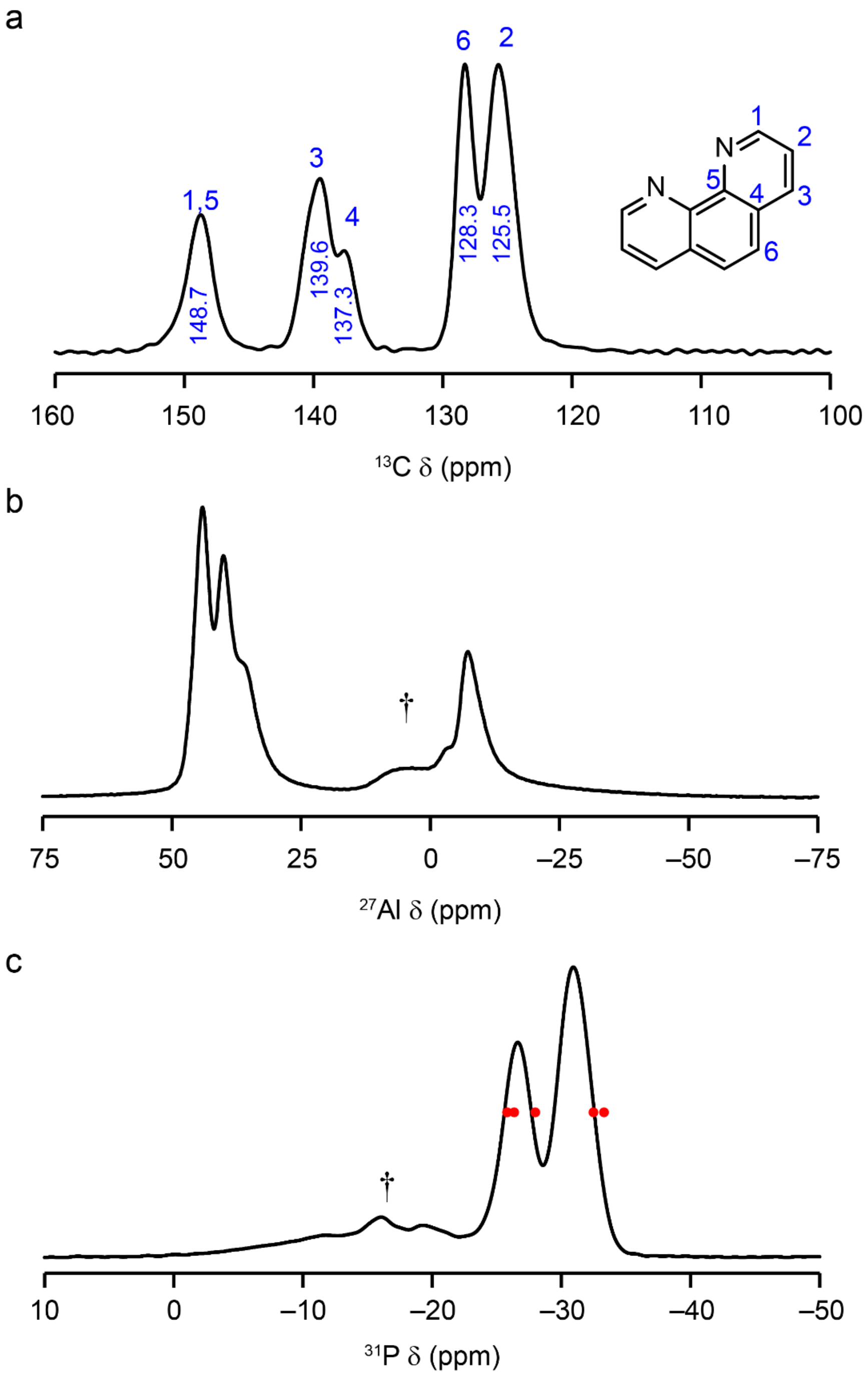

Figure S8. Solid-state NMR spectra of as-prepared STA-28: (a) ^{13}C (12.5 kHz CP MAS); (b) ^{27}Al (14 kHz MAS); (c) ^{31}P (14 kHz MAS), red points indicate the chemical shifts predicted by the simulation program DISCO[S21] for the experimental crystal structure). Signals from amorphous impurities (Al oxyhydroxides and phosphates) are denoted with a dagger (†).

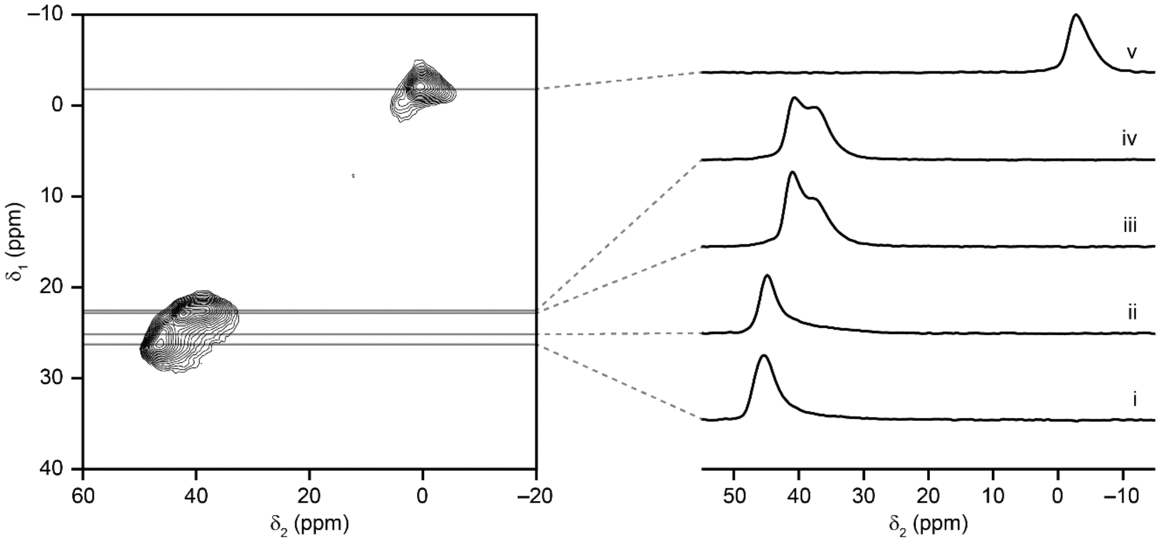

Figure S9. ^{27}Al MQMAS NMR spectrum of as-prepared STA-28, with extracted rows corresponding to each of the five Al signals identified. Note that signals iii and iv overlap in δ_1, such that their corresponding rows contain intensity from both signals.

S6. Structural comparison of STA-28

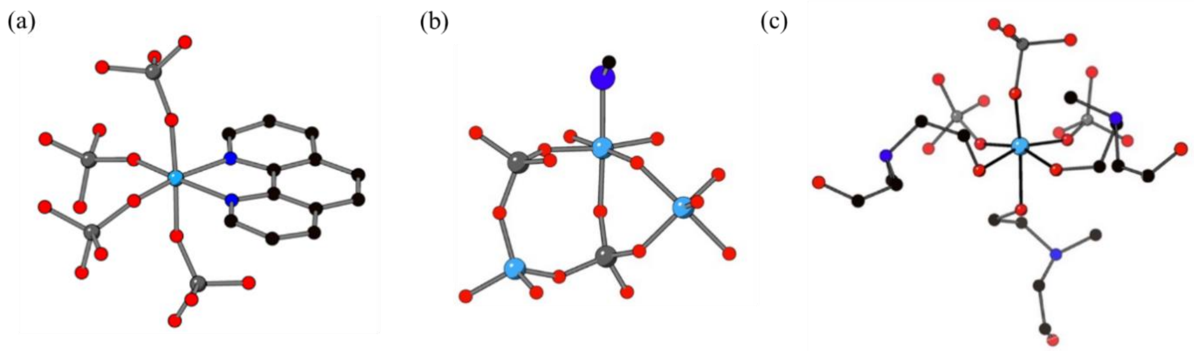

Figure S10. Comparison of Al coordination of framework-bound templates in AlPOs**.** (a) octahedral unit of STA-28, Al bound to 2 N from 1,10-phenanthroline and 4 framework O, (b) octahedral unit of IST-1, [S22] Al bound to a N of methylamine and 5 O, (c) octahedral unit of ECR-40,[S23] Al atom bound to 6 O atoms, one each from three tris(2-hydroxyethyl)methylammonium molecules and a further three linked to P. [red = O, grey = P, light blue = Al, black = C, dark blue = N]. For ECR-40, template removal results in a tetrahedral framework with Al-O-Al bonds.[S23]

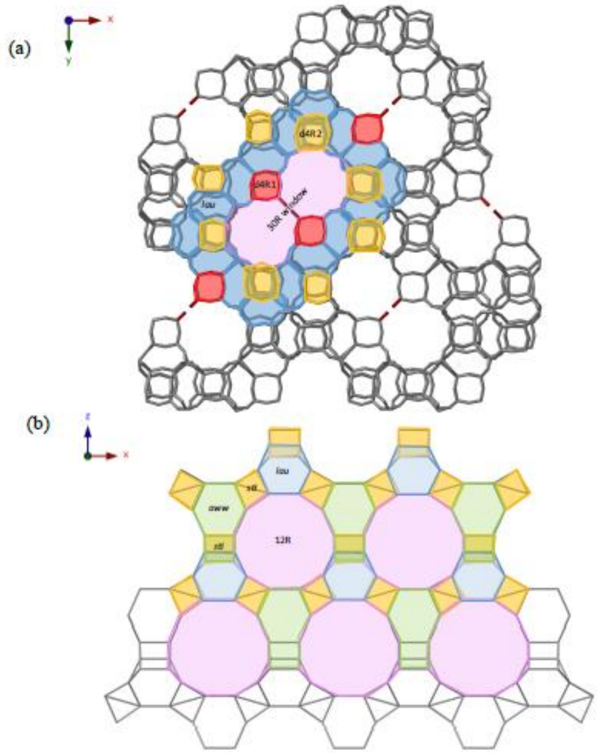

Figure S11. The building units present within (a) ITQ-37 (ITV) and (b) STA-1 (SAO). ITQ- 37 is the most similar in structure to STA-28 with two types of d4R linked by t-*lau*. Notably, the -ITV framework is not fully connected as a *d4r* terminated by a hydroxyl faces a cavity. STA-28 is characterised by a framework density (T-atoms per 1000 Å^3) of 15.6. This makes it denser than the structures of STA-1 and ITQ-37, which have framework densities of 14.2 and 10.3 T/1000 Å^3, respectively.

S7. Rietveld refinements of AlPOs prepared with other phenanthrolines

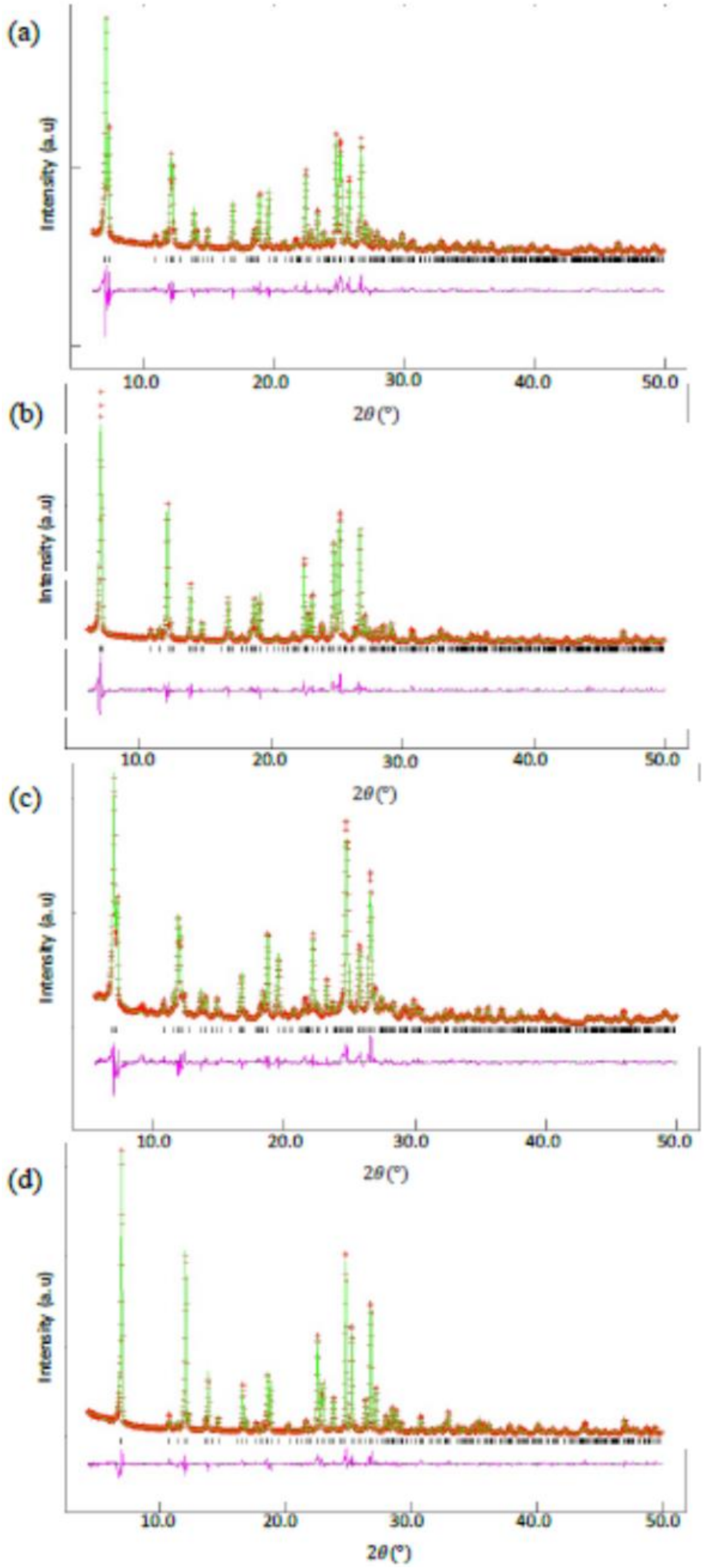

Figure S12. Rietveld refinement for STA-28 prepared using (a) 4-methyl phen, *Rwp* = 9.38 %, (b) 5-methyl phen, *Rwp* = 10.58 %, (c) 4,7-dimethyl phen, *Rwp* = 9.49 % and (d) 5,6-dimethyl phen, *Rwp* = 9.63 %.

Table S7. Unit cell parameters obtained from Rietveld analysis of STA-28 prepared using methylated phenanthrolines, where phen = 1,10-phenanthroline. Upon increasing size of the OSDA, the unit cell gets slightly larger.

OSDA used	*a* (Å)	*b*(Å)	*c* (Å)	β (°)	Volume (Å^3)
phen	13.9916(5)	25.4790(9)	14.4450(5)	95.9949(30)	5121.4(4)
4-methyl-phen	13.9168(16)	25.6501(29)	14.4538(15)	96.165(4)	5129.7(17)
5-methyl-phen	14.1887(8)	25.4482(13)	14.4083(7)	95.340(4)	5179.9(7)
4,7-dimethyl-phen	13.8916(21)	25.910(4)	14.4798(20)	96.340(7)	5179.8(21)
5,6-dimethyl-phen	14.3682(9)	25.3529(9)	14.3687(8)	95.112(4)	5213.4(4)

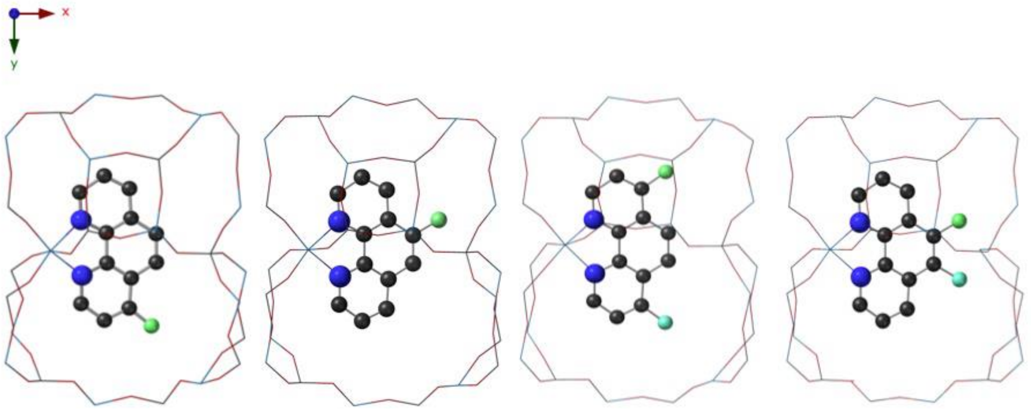

Figure S13. The refined positions of the methylated phenanthroline molecules within the 12R channels of STA-28, viewed along z. [black = C atom, blue = N, green and cyan = C methyl group]

Table S8. Fractional atomic coordinates and occupancies from the refinement of STA-28 prepared with 4-methyl phenanthroline. (U_{iso} = 0.002 Å^2 for the framework atoms & 0.001 Å^2 for all C & N).

Atom	Fractional Coordinates			Fractional Occupancy
	x	y	z	
**P1**	0.3558(18)	0.2887(11)	0.6240(19)	1.00
**P2**	0.1299(21)	0.1630(10)	0.5821(19)	1.00
**P3**	0.0311(19)	0.3684(11)	0.7051(18)	1.00
**P4**	0.1253(21)	0.5889(10)	0.6017(19)	1.00
**P5**	0.3600(20)	0.4558(11)	0.6296(19)	1.00
**Al1**	0.3515(21)	0.1652(11)	0.6180(22)	1.00
**Al2**	0.1284(23)	0.2785(11)	0.5917(21)	1.00
**Al3**	0.1298(22)	0.4550(11)	0.5921(20)	1.00
**Al4**	0.3525(21)	0.5867(10)	0.6317(21)	1.00
**Al5**	0.5010(14)	0.3739(9)	0.7590(18)	1.00
**O1**	0.4309(21)	0.3166(14)	0.6952(25)	1.00
**O2**	0.2473(23)	0.2972(18)	0.6231(32)	1.00
**O3**	0.3918(31)	0.2945(18)	0.5269(23)	1.00
**O4**	0.3785(30)	0.2310(11)	0.6374(34)	1.00
**O5**	0.2311(23)	0.1574(17)	0.6206(33)	1.00
**O6**	0.0904(30)	0.2155(12)	0.6045(33)	1.00
**O7**	0.0592(27)	0.1360(18)	0.6328(24)	1.00
**O8**	0.0961(29)	0.1546(18)	0.4820(22)	1.00
**O9**	0.0651(29)	0.3186(14)	0.6576(31)	1.00
**O10**	0.0725(30)	0.4150(15)	0.6611(31)	1.00

**O11**	0.0727(29)	0.3699(16)	0.8067(20)	1.00
**O12**	-0.0786(22)	0.3696(17)	0.7034(25)	1.00
**O13**	0.1203(33)	0.5256(11)	0.6102(33)	1.00
**O14**	0.2521(23)	0.4440(17)	0.621(4)	1.00
**O15**	0.2321(22)	0.6027(15)	0.6317(33)	1.00
**O16**	0.1290(34)	0.5971(16)	0.4911(21)	1.00
**O17**	0.0680(26)	0.6317(16)	0.6511(25)	1.00
**O18**	0.3707(31)	0.5174(11)	0.6505(30)	1.00
**O19**	0.4206(21)	0.4276(13)	0.7077(24)	1.00
**O20**	0.3827(33)	0.4454(19)	0.5302(22)	1.00
**N1**	0.60596(32)	0.32846(32)	0.80566(32)	1.00
**N10**	0.60266(32)	0.43156(32)	0.81576(32)	1.00
**C1A**	0.68846(32)	0.35146(32)	0.84706(32)	1.00
**C2**	0.60266(32)	0.27606(32)	0.80546(32)	1.00
**C3**	0.68006(32)	0.24526(32)	0.84496(32)	1.00
**C4**	0.76226(32)	0.26776(32)	0.88376(32)	1.00
**C5**	0.85206(32)	0.35296(32)	0.92426(32)	1.00
**C5A**	0.76876(32)	0.32376(32)	0.88536(32)	1.00
**C6**	0.85196(32)	0.40596(32)	0.92256(32)	1.00
**C6A**	0.76826(32)	0.43526(32)	0.88746(32)	1.00
**C7**	0.76296(32)	0.49066(32)	0.88786(32)	1.00
**C8**	0.67926(32)	0.51446(32)	0.85466(32)	1.00
**C9**	0.59816(32)	0.48366(32)	0.82066(32)	1.00
**C10A**	0.68636(32)	0.40806(32)	0.85046(32)	1.00
**CMe1**	0.726(12)	0.491(7)	0.2080(27)	0.00(34)
**CMe2**	0.65941(32)	0.22941(32)	0.08061(32)	1.00(34)

Table S9. Fractional atomic coordinates and occupancies from the refinement of STA-28 prepared with 5-methyl phenanthroline. (U_{iso} = 0.002 $Å^2$ for the framework atoms & 0.001 $Å^2$ for all C & N atoms).

**Atom**	**Fractional Coordinates**			**Fractional Occupancy**
	**x**	**y**	**z**	
**P1**	0.3548(18)	0.2934(11)	0.6277(20)	1.00
**P2**	0.1371(19)	0.1600(10)	0.5989(19)	1.00
**P3**	0.0294(18)	0.3718(13)	0.7048(17)	1.00
**P4**	0.1298(20)	0.5855(10)	0.5969(19)	1.00
**P5**	0.3564(19)	0.4586(11)	0.6312(19)	1.00
**Al1**	0.3541(19)	0.1657(11)	0.6208(21)	1.00
**Al2**	0.1312(24)	0.2875(12)	0.5881(20)	1.00
**Al3**	0.1287(21)	0.4613(12)	0.5876(20)	1.00
**Al4**	0.3499(21)	0.5847(10)	0.6266(22)	1.00
**Al5**	0.4964(13)	0.3771(11)	0.7557(19)	1.00
**O1**	0.4274(22)	0.3235(15)	0.6917(25)	1.00
**O2**	0.2464(24)	0.3002(19)	0.6346(29)	1.00
**O3**	0.3760(30)	0.3008(20)	0.5283(23)	1.00
**O4**	0.3770(28)	0.2333(12)	0.6379(35)	1.00
**O5**	0.2392(22)	0.1478(15)	0.6298(29)	1.00
**O6**	0.1209(33)	0.2204(12)	0.6048(32)	1.00
**O7**	0.0648(27)	0.1235(20)	0.6363(23)	1.00
**O8**	0.1178(29)	0.1497(17)	0.4915(21)	1.00
**O9**	0.0550(29)	0.3221(14)	0.6519(32)	1.00
**O10**	0.0701(29)	0.4190(15)	0.6572(32)	1.00
**O11**	0.0701(26)	0.3734(16)	0.8086(21)	1.00
**O12**	-0.0808(22)	0.3734(19)	0.7006(25)	1.00
**O13**	0.1061(32)	0.5267(12)	0.6111(31)	1.00
**O14**	0.2488(23)	0.4502(20)	0.6231(30)	1.00
**O15**	0.2321(21)	0.5957(16)	0.6311(30)	1.00

**O16**	0.1340(32)	0.5971(17)	0.4915(22)	1.00
**O17**	0.0644(26)	0.6198(20)	0.6505(25)	1.00
**O18**	0.3706(31)	0.5176(12)	0.6444(31)	1.00
**O19**	0.4165(21)	0.4314(15)	0.7124(23)	1.00
**O20**	0.3844(29)	0.4502(20)	0.5324(23)	1.00
**N1**	0.6042(5)	0.3267(5)	0.8039(5)	1.00
**N10**	0.6009(5)	0.4298(5)	0.8140(5)	1.00
**C1A**	0.6867(5)	0.3497(5)	0.8453(5)	1.00
**C2**	0.6009(5)	0.2743(5)	0.8037(5)	1.00
**C3**	0.6783(5)	0.2435(5)	0.8432(5)	1.00
**C4**	0.7605(5)	0.2660(5)	0.8820(5)	1.00
**C5**	0.8503(5)	0.3512(5)	0.9225(5)	1.00
**C5A**	0.7670(5)	0.3220(5)	0.8836(5)	1.00
**C6**	0.8502(5)	0.4042(5)	0.9208(5)	1.00
**C6A**	0.7665(5)	0.4335(5)	0.8857(5)	1.00
**C7**	0.7612(5)	0.4889(5)	0.8861(5)	1.00
**C8**	0.6775(5)	0.5127(5)	0.8529(5)	1.00
**C9**	0.5964(5)	0.4819(5)	0.8189(5)	1.00
**C10A**	0.6846(5)	0.4063(5)	0.8487(5)	1.00
**CMe1**	0.9369(5)	0.4329(5)	0.9619(5)	1.00(6)
**CMe2**	0.9379(5)	0.3219(5)	0.9629(5)	0.00(6)

Table S10. Fractional atomic coordinates and occupancies from the refinement of STA-28 prepared with 4,7-dimethyl-1,10-phenanthroline. (U_{iso} = 0.002 Å^2 for framework atoms & 0.001 Å^2 for C & N).

**Atom**	**Fractional Coordinates**			**Fractional Occupancy**
	**x**	**y**	**z**	
**P1**	0.3642(20)	0.2895(10)	0.6310(22)	1.00
**P2**	0.1142(22)	0.1596(11)	0.5946(22)	1.00
**P3**	0.0286(21)	0.3776(12)	0.7068(19)	1.00
**P4**	0.1343(24)	0.5788(10)	0.5938(20)	1.00
**P5**	0.3611(21)	0.4588(11)	0.6259(21)	1.00
**Al1**	0.3530(22)	0.1620(11)	0.6218(22)	1.00
**Al2**	0.1246(23)	0.2865(11)	0.5918(22)	1.00
**Al3**	0.1356(24)	0.4621(13)	0.5935(23)	1.00
**Al4**	0.3572(25)	0.5804(11)	0.6335(24)	1.00
**Al5**	0.5029(16)	0.3798(09)	0.7489(20)	1.00
**O1**	0.4232(24)	0.3277(14)	0.6908(28)	1.00
**O2**	0.2510(23)	0.2999(19)	0.6260(40)	1.00
**O3**	0.3957(34)	0.2996(21)	0.5293(24)	1.00
**O4**	0.3770(34)	0.2291(11)	0.643(4)	1.00
**O5**	0.2259(23)	0.1516(16)	0.621(4)	1.00
**O6**	0.0987(34)	0.2207(12)	0.603(4)	1.00
**O7**	0.0575(29)	0.1176(17)	0.6384(26)	1.00
**O8**	0.1050(40)	0.1511(18)	0.4881(23)	1.00
**O9**	0.0703(34)	0.3347(15)	0.6498(32)	1.00
**O10**	0.0606(32)	0.4262(16)	0.6579(34)	1.00
**O11**	0.0804(33)	0.3809(16)	0.8069(23)	1.00
**O12**	-0.0822(24)	0.3773(18)	0.7054(28)	1.00
**O13**	0.1100(40)	0.5243(12)	0.6166(32)	1.00
**O14**	0.2539(25)	0.4476(19)	0.6290(40)	1.00
**O15**	0.2376(24)	0.5896(16)	0.6310(40)	1.00
**O16**	0.1240(40)	0.5895(16)	0.4863(22)	1.00
**O17**	0.0677(31)	0.6073(18)	0.6454(28)	1.00
**O18**	0.3756(34)	0.5265(12)	0.6540(35)	1.00
**O19**	0.4221(24)	0.4356(14)	0.7041(27)	1.00
**O20**	0.3840(40)	0.4472(180	0.5268(250	1.00
**N1**	0.6026(3)	0.3251(3)	0.8023(3)	1.00

**N10**	0.5993(3)	0.4282(3)	0.8124(3)	1.00
**C1A**	0.6851(3)	0.3481(3)	0.8437(3)	1.00
**C2**	0.5993(3)	0.2727(3)	0.8021(3)	1.00
**C3**	0.6767(3)	0.2419(3)	0.8416(3)	1.00
**C4**	0.7589(3)	0.2644(3)	0.8804(3)	1.00
**C5**	0.8487(3)	0.3496(3)	0.9209(3)	1.00
**C5A**	0.7654(3)	0.3204(3)	0.8820(3)	1.00
**C6**	0.8486(3)	0.4026(3)	0.9192(3)	1.00
**C6A**	0.7649(3)	0.4319(3)	0.8841(3)	1.00
**C7**	0.7596(3)	0.4873(3)	0.8845(3)	1.00
**C8**	0.6759(3)	0.5111(3)	0.8513(3)	1.00
**C9**	0.5948(3)	0.4803(3)	0.8173(3)	1.00
**C10A**	0.6830(3)	0.4047(3)	0.8471(3)	1.00
**CMe1**	0.6593(3)	0.5193(3)	0.0813(3)	1.00
**CMe2**	0.6613(3)	0.2313(3)	0.0825(3)	1.00

Table S11. Fractional atomic coordinates and occupancies from the refinement of STA-28 prepared with 5,6-dimethyl-1,10-phenanthroline. (U_{iso} = 0.002 Å^2 for framework atoms & 0.001 Å^2 for C & N).

**Atom**	**Fractional Coordinates**			**Fractional Occupancy**
	**x**	**y**	**z**	
**P1**	0.3504(18)	0.2923(13)	0.6334(21)	1.0
**P2**	0.1351(24)	0.1584(14)	0.5989(22)	1.0
**P3**	0.0316(20)	0.3716(16)	0.7139(21)	1.0
**P4**	0.1335(23)	0.5869(14)	0.5972(22)	1.0
**P5**	0.3571(20)	0.4567(12)	0.6361(20)	1.0
**Al1**	0.3506(21)	0.1641(14)	0.6247(25)	1.0
**Al2**	0.1284(23)	0.2890(19)	0.5835(22)	1.0
**Al3**	0.1319(23)	0.4603(17)	0.5874(22)	1.0
**Al4**	0.3493(22)	0.5855(12)	0.6284(23)	1.0
**Al5**	0.4961(16)	0.3761(11)	0.7488(22)	1.0
**O1**	0.4275(25)	0.3220(15)	0.6892(29)	1.00
**O2**	0.2444(22)	0.2999(22)	0.6235(33)	1.00
**O3**	0.3690(32)	0.2988(29)	0.5327(23)	1.00
**O4**	0.3730(30)	0.2323(14)	0.6400(40)	1.00
**O5**	0.2359(24)	0.1473(21)	0.6356(31)	1.00
**O6**	0.1180(40)	0.2204(17)	0.5990(40)	1.00
**O7**	0.0627(31)	0.1230(25)	0.6381(26)	1.00
**O8**	0.1398(33)	0.1478(21)	0.4927(24)	1.00
**O9**	0.0493(29)	0.3248(19)	0.6455(35)	1.00
**O10**	0.0764(33)	0.4217(20)	0.6710(40)	1.00
**O11**	0.0745(33)	0.3746(19)	0.8120(23)	1.00
**O12**	-0.0780(23)	0.3740(21)	0.6974(30)	1.00
**O13**	0.1190(40)	0.5270(160	0.6070(40)	1.00
**O14**	0.2510(22)	0.4484(23)	0.6170(40)	1.00
**O15**	0.2327(21)	0.5952(21)	0.6432(33)	1.00
**O16**	0.1220(40)	0.5968(19)	0.4895(23)	1.00
**O17**	0.0698(27)	0.6245(22)	0.6470(29)	1.00
**O18**	0.3700(40)	0.5171(13)	0.6450(40)	1.00
**O19**	0.4202(21)	0.4324(15)	0.7124(27)	1.00
**O20**	0.3842(29)	0.4536(27)	0.5342(23)	1.00
**N1**	0.6014(7)	0.3239(7)	0.8011(7)	1.00
**N10**	0.5981(7)	0.4270(7)	0.8112(7)	1.00
**C1A**	0.6839(7)	0.3469(7)	0.8425(7)	1.00
**C2**	0.5980(7)	0.2714(7)	0.8008(7)	1.00
**C3**	0.6754(7)	0.2406(7)	0.8403(7)	1.00
**C4**	0.7577(7)	0.2632(7)	0.8792(7)	1.00
**C5**	0.8475(7)	0.3484(7)	0.9197(7)	1.00

**C5A**	0.7642(7)	0.3192(7)	0.8808(7)	1.00
**C6**	0.8474(7)	0.4014(7)	0.9180(7)	1.00
**C6A**	0.7637(7)	0.4307(7)	0.8829(7)	1.00
**C7**	0.7584(7)	0.4861(7)	0.8833(7)	1.00
**C8**	0.6747(7)	0.5099(7)	0.8501(7)	1.00
**C9**	0.5939(7)	0.4791(7)	0.8161(7)	1.00
**C10A**	0.6818(7)	0.4035(7)	0.8459(7)	1.00
**CMe1**	0.9341(7)	0.4301(7)	0.9591(7)	1.0
**CMe2**	0.9351(7)	0.3191(7)	0.9601(7)	1.0

S8. Synthesis and analysis of FeAlPO STA-28

In attempts to replace Al in the STA-28 $AlPO_4$ structure, $Al(OH)_3$ was substituted with Fe(II) acetate in the gel preparations, up to an Fe/P ratio of 0.3, while 1,10-phenanthroline was kept in excess of the added Fe. Powder X-ray diffraction patterns, image of the as-made FeAlPO samples and SEM images of FeAlPO STA-28 prepared with Fe/P ratio of 0.20 are shown in Figure S14.

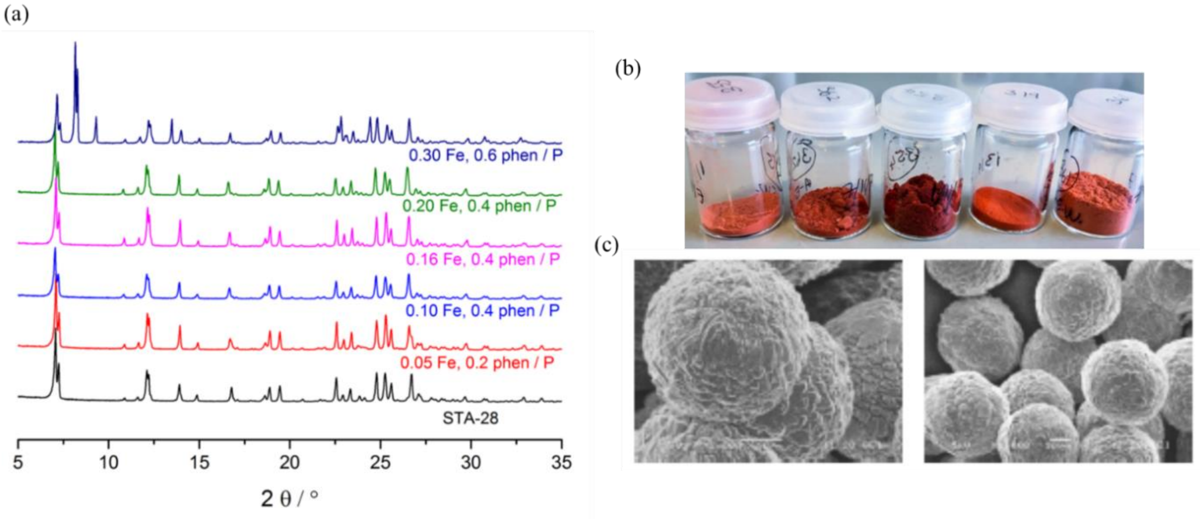

Figure S14. (a) Powder X-ray diffraction patterns for as-made FeAlPO STA-28 prepared with 1,10-phenanthroline and using different Fe/P ratios in the gel. From upper to lower, the patterns represent FeAlPO STA-28 prepared with Fe/P ratio of: (i) 0.30 (ii) 0.20; (iii) 0.16; (iv) 0.10; (v) 0.05 and (vi) 0.0 (AlPO STA-28). (b) Image of the as-made FeAlPO samples, from left to right; 0.05 Fe/P, 0.10 Fe/P, 0.16 Fe/P, 0.20 Fe/P and 0.30 Fe/P. (c) SEM images of FeAlPO STA-28 prepared with Fe/P ratio of 0.20.

XRF analysis on the sample prepared with $(Fe/P)_{gel}$ = 0.2 gives an empirical framework formula of $Fe_{0.78}Al_{4.22}P_5O_{20}$. FeAlPO STA-28 was also obtained if ferrous $Fe(OAc)_2$ was replaced with $FeCl_3$ in the gel (Figure S15).

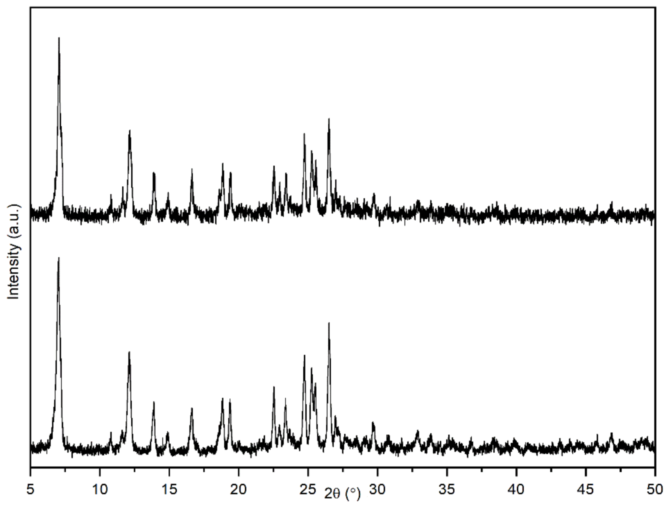

Figure S15. FeAlPO STA-28 prepared using Fe(II) acetate (bottom) and Fe(III) chloride (top) in the gel ($(Fe/P)_{gel}$ = 0.2)

.

S9. Spectroscopy of FeAlPO STA-28 (Fe/P 0.20 in gel, 0.155 in product)

UV-visible

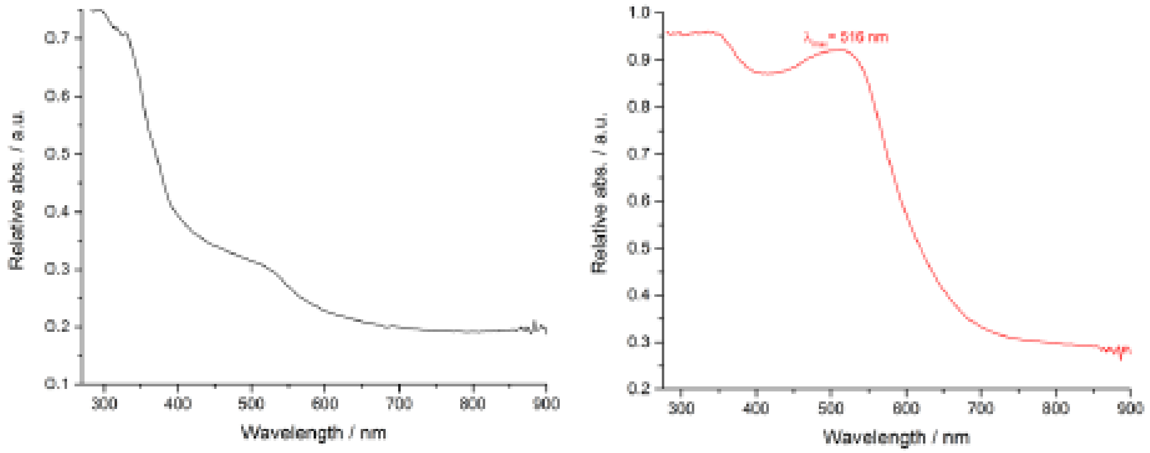

Figure S16. Solid-state UV-vis absorption spectra for AlPO (left) and FeAlPO (right) STA-28.

EPR

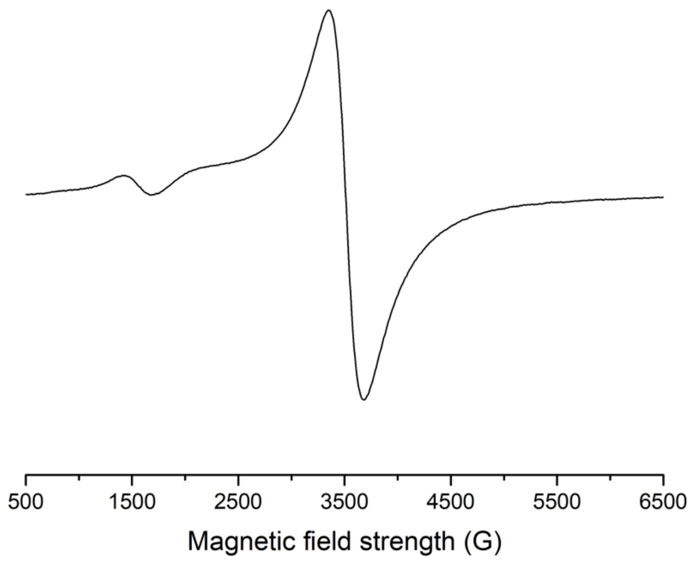

Figure S17. EPR spectrum for FeAlPO STA-28

Mössbauer

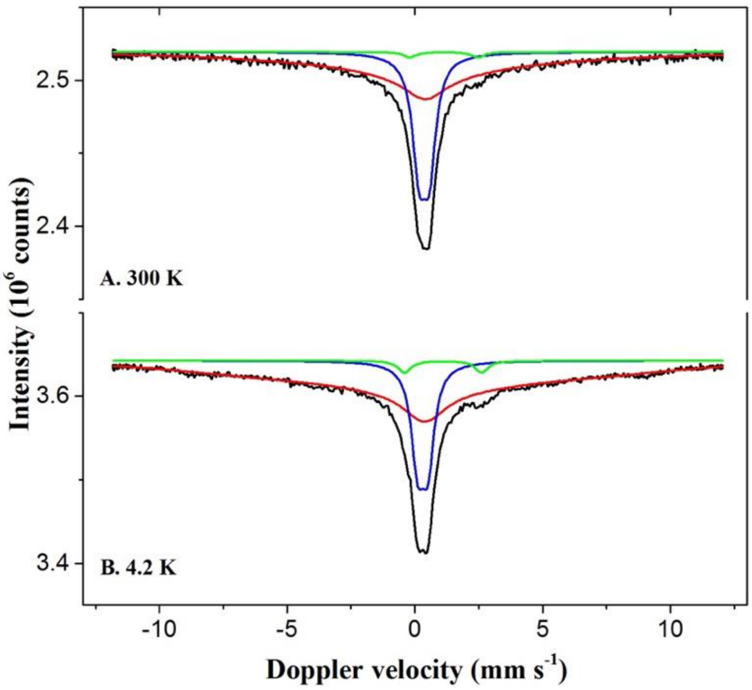

Figure S18. Mössbauer spectra (black lines) obtained at 300 and 4.2 K for FeAlPO STA-28. Fitted lines are given in colour.

Table S12. Mössbauer parameters of FeAlPO STA-28 derived from spectral fitting in Figure 23.

Temperature (K)	IS (mm s^{-1})	QS (mm s^{-1})	τ_R (s)	Γ (mm s^{-1})	Iron Species	Fit line (Fig. S23)	Spectral Contribution (%)
300	0.36	0.40	-	0.62	Fe^{3+}	Red	35
	0.41	-	1.8 10^{-9}	1.61	Fe^{3+}	Blue	63
	1.13	2.68	-	0.55	Fe^{2+}	Green	2
4.2	0.31	0.40	-	0.60	Fe^{3+}	Red	22
	0.39	-	3.0 10^{-9}	1.61	Fe^{3+}	Blue	75
	1.11	3.0	-	0.59	Fe^{2+}	Green	3

Experimental uncertainties: isomer shift: IS ± 0.02mm s^{-1}; quadrupole splitting: QS ± 0.02 mm s^{-1}; line width: Γ ± 0.02 mm s^{-1}; relaxation time: τ_R ± 2%; spectral contribution: ±2%.

Solid State NMR

Figure S19a shows the ^{27}Al NMR spectra of FeAPO STA-28 prepared with Fe/P gel ratios of 0.05, 0.10, 0.16 and 0.20 and Figure S24b shows a plot of the ratio of tetrahedral/octahedral Al ($Al^{[IV]}/Al^{[VI]}$) against Fe/P. As the Fe content of the material increases, the ^{27}Al resonances broaden owing to both increased disorder and paramagnetic-induced nuclear relaxation enhancement. Adding more Fe appears to reduce the amount of tetrahedral Al preferentially. Given the results of the other characterisation techniques presented in the main text, which show that Fe substitutes almost exclusively on the octahedral Al5 site, this appears counter-intuitive. A likely explanation is that the paramagnetic Fe^{3+} is generates a "sphere of invisibility", whereby paramagnetic relaxation effects result in signals from ^{27}Al near to the Fe being broadened beyond detection with the current NMR experiments (any through-bond hyperfine effects resulting in paramagnetic shifts are expected to be small as the shortest bonded pathway from Al to Fe is Al-O-P-O-Fe). The relaxation effect would be more pronounced on the $Al^{[IV]}$ sites, since there are many more of these in close proximity to any given octahedral site - there are 12 $Al^{[IV]}$ sites within 5.2 – 6.2 Å of a $Fe^{[VI]}$ while the nearest $Al^{[VI]}$ would be further than 9 Å away. Substituting any one octahedral site with Fe completely removes the signal from one $Al^{[VI]}$ but would also significantly reduce (or remove) the signals from many nearby $Al^{[IV]}$ sites. This suggestion is supported by the fact that the resonance lines can be seen to broaden significantly as the Fe content increases, and also the total signal intensity per transient drops significantly with increasing Fe content (for the 0.05 Fe/P sample, the spectrum shown was recorded with 128 transients and moderate receiver gain, whereas for the 0.2 Fe/P sample 4096 transients were required with maximum receiver gain). The observation of some octahedral Al even with Fe/P = 0.2 is in agreement with the Rietveld-refined Fe occupancy of 0.73.

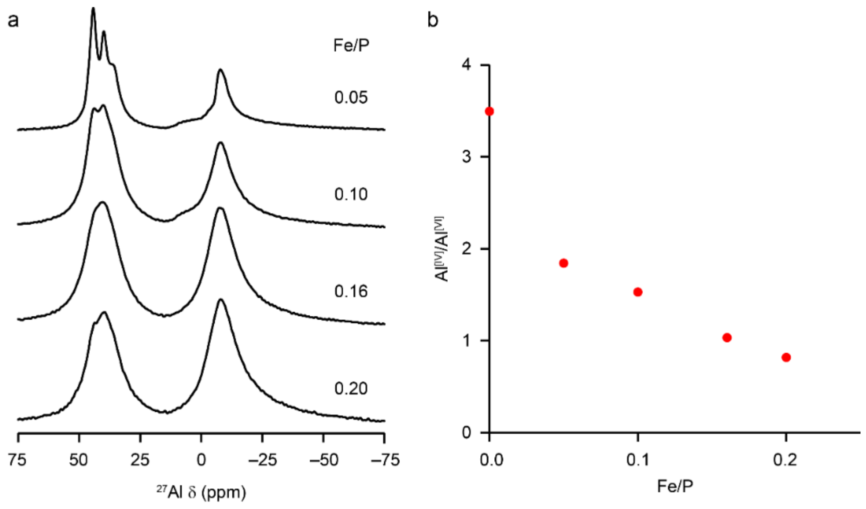

Figure S19. (a) ^{27}Al (9.4 T, 14 kHz MAS) NMR spectra of FeAPO STA-28 with the indicated Fe/P ratios. (b) Plot of $Al^{[IV]}/Al^{[VI]}$ against Fe/P.

S10. Rietveld refinement on FeAlPO STA-28

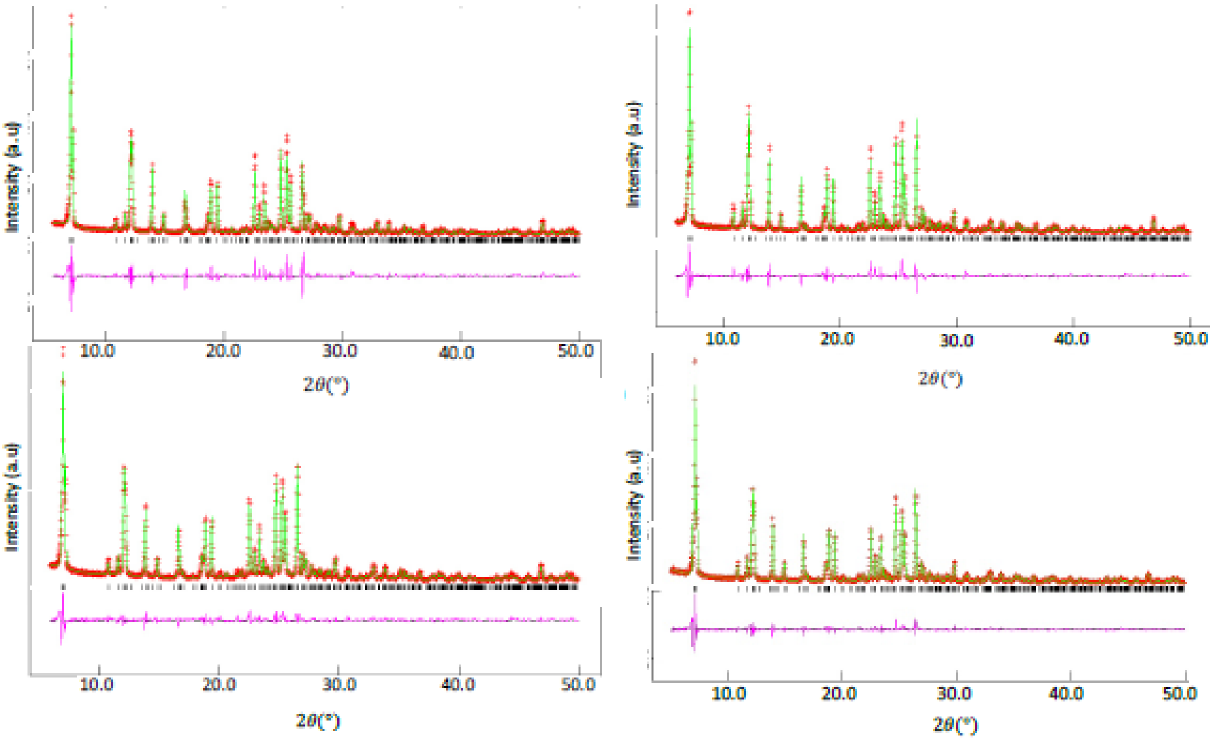

Figure S20. Rietveld refinement on the space group *I* 2/*a* for FeAlPO STA-28 prepared using Fe/P ratio (top left) 0.05, R_{wp} = 11.7 %, (bottom left) 0.10, R_{wp} = 6.91 %, (top right) 0.16, R_{wp} = 7.66 % and (bottom right) 0.20, R_{wp} = 3.7 %.

Table S13. Unit cell parameters obtained from Rietveld analysis of FeAlPO STA-28.

OSDA used	Fe/P in starting gel	Fe occupancy in Octahedral Site	*a* (Å)	*b*(Å)	*c* (Å)	β (°)	Volume (Å^3)
1,10-phen	Fe 0.05	0.25(4)	14.0088(18)	25.4386(32)	14.4543(17)	96.424(6)	5118.7(18)
	Fe 0.10	0.42(2)	14.0149(7)	25.4385(12)	14.4651(6)	96.518(4)	5123.7(6)
	Fe 0.16	0.55(3)	14.0341(7)	25.4432(12)	14.4790(6)	96.653(4)	5135.3(5)
	Fe 0.20	0.73(1)	14.0495(3)	25.4621(6)	14.4890(3)	96.7225(2)	5149.1(5)
4-methyl-phen	Fe 0.20	0.479(31)	13.9927(8)	25.6053(31)	14.4700(17)	96.847(5)	5147.5(18)

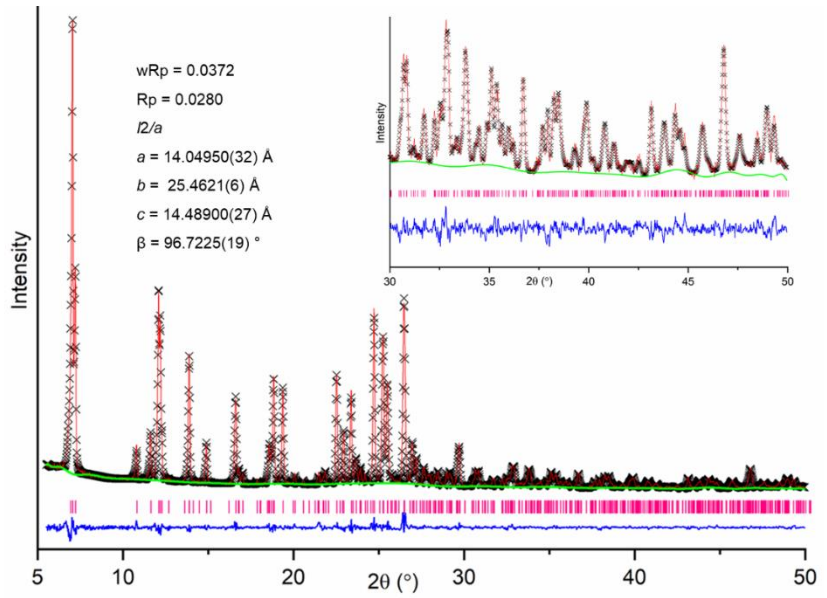

Figure S21. Restrained Rietveld refinement of as-made, hydrated FeAlPO STA-28 (Fe/P=0.155) in space group *I* 2/*a* using the AlPO structure determined by single crystal as a starting model.

Table S14. Crystallographic details obtained from Rietveld refinement against PXRD data for as-made FeAlPO STA-28 (Fe/P=0.155).

**FeAlPO as-prepared**	
**OSDA used**	1,10-phenanthroline
**Unit cell**	$C_{12}Al_{4.27}Fe_{0.73}N_2O_{20}\ P_5$
**Temperature/K**	298
**Space group**	*I*2/*a*
**X-ray source**	Cu
**Diffractometer**	Stoe
**Wavelength (Å)**	1.54056
**a/ Å**	14.04950(32)
**b/ Å**	25.4621(6)
**c/ Å**	14.48900(27)
**Volume/Å^3**	5147.52(25)
**R_p**	0.0280
**R_{wp}**	0.0372
**χ^2**	3.863

Table S15. Fractional atomic coordinates, occupancies and U_{iso} parameters obtained from Rietveld refinement against PXRD data for as-made FeAlPO STA-28.

Atom	x	y	z	Occup.	Multipl.	Uiso
**P1**	0.3584(10)	0.2878(6)	0.6296(10)	1.0	8	0.01525
**P2**	0.1257(9)	0.1608(5)	0.5876(10)	1.0	8	0.01525
**P3**	0.0323(8)	0.3745(7)	0.6984(8)	1.0	8	0.01525
**P4**	0.1335(10)	0.5866(5)	0.5926(10)	1.0	8	0.01525
**P5**	0.3586(10)	0.4604(6)	0.6320(9)	1.0	8	0.01525
**Al1**	0.3524(10)	0.1634(5)	0.6252(10)	1.0	8	0.01525
**Al2**	0.1346(10)	0.2851(6)	0.5853(12)	1.0	8	0.01525
**Al3**	0.1381(10)	0.4607(6)	0.5835(11)	1.0	8	0.01525
**Al4**	0.3503(10)	0.5847(6)	0.6270(10)	1.0	8	0.01525
**Al5**	0.4992(6)	0.3762(8)	0.7500(7)	0.268(12)	8	0.01525
**Fe1**	0.4992(6)	0.3762(8)	0.7500(7)	0.732(12)	8	0.01525
**O1**	0.4222(13)	0.3199(8)	0.7002(11)	1.0	8	0.01525
**O2**	0.2510(8)	0.2978(9)	0.6281(18)	1.0	8	0.01525
**O3**	0.3848(18)	0.3004(9)	0.5300(9)	1.0	8	0.01525
**O4**	0.3767(18)	0.2277(4)	0.6474(17)	1.0	8	0.01525
**O5**	0.2323(8)	0.1508(9)	0.6244(16)	1.0	8	0.01525
**O6**	0.1069(16)	0.2203(4)	0.5995(18)	1.0	8	0.01525
**O7**	0.0548(13)	0.1274(8)	0.6335(10)	1.0	8	0.01525
**O8**	0.1128(16)	0.1479(9)	0.4820(9)	1.0	8	0.01525
**O9**	0.0618(13)	0.3244(7)	0.6451(13)	1.0	8	0.01525
**O10**	0.0645(13)	0.4226(7)	0.6436(13)	1.0	8	0.01525
**O11**	0.0841(12)	0.3755(7)	0.7962(10)	1.0	8	0.01525
**O12**	-0.0765(9)	0.3759(7)	0.6988(11)	1.0	8	0.01525
**O13**	0.1163(18)	0.5265(4)	0.6046(17)	1.0	8	0.01525
**O14**	0.2523(8)	0.4465(9)	0.6268(20)	1.0	8	0.01525
**O15**	0.2366(8)	0.6000(9)	0.6347(17)	1.0	8	0.01525
**O16**	0.1282(19)	0.5983(9)	0.4863(8)	1.0	8	0.01525
**O17**	0.0647(11)	0.6207(7)	0.6415(13)	1.0	8	0.01525
**O18**	0.3708(18)	0.5202(4)	0.6507(16)	1.0	8	0.01525
**O19**	0.4182(14)	0.4295(8)	0.7070(11)	1.0	8	0.01525
**O20**	0.3864(18)	0.4491(10)	0.5328(9)	1.0	8	0.01525
**N1**	0.6138(32)	0.3222(17)	0.809(4)	1.0	8	0.01525
**N10**	0.6140(29)	0.4312(17)	0.821(4)	1.0	8	0.01525
**C1A**	0.690(5)	0.3474(15)	0.852(6)	1.0	8	0.01525
**C2**	0.605(4)	0.2715(19)	0.805(4)	1.0	8	0.01525
**C3**	0.680(5)	0.2393(15)	0.848(5)	1.0	8	0.01525
**C4**	0.7664(35)	0.2624(26)	0.885(4)	1.0	8	0.01525
**C5**	0.857(4)	0.3465(20)	0.922(6)	1.0	8	0.01525
**C5A**	0.774(4)	0.3184(27)	0.884(6)	1.0	8	0.01525
**C6**	0.858(4)	0.4006(20)	0.924(6)	1.0	8	0.01525
**C6A**	0.7766(34)	0.4286(26)	0.887(5)	1.0	8	0.01525
**C7**	0.7801(29)	0.4846(27)	0.888(4)	1.0	8	0.01525
**C8**	0.695(5)	0.5106(14)	0.855(5)	1.0	8	0.01525
**C9**	0.609(4)	0.4826(18)	0.824(4)	1.0	8	0.01525
**C10A**	0.690(4)	0.4021(14)	0.855(6)	1.0	8	0.01525

Table S16. T-O, C-N/C-C bond distances (Å) and O-T-O/T-O-T angles (°) obtained from Rietveld refinement against PXRD data for as-made FeAlPO STA-28.

T-O, C-N/C-C bond distances (Å) and O-T-O/T-O-T angles					
**P1 O1**	1.518(13)	**N1 C1A**	1.337(17)	**O9 P3 O10**	106.2(10)
**P1 O2**	1.527(14)	**N1 C2**	1.296(17)	**O9 P3 O11**	110.4(12)
**P1 O3**	1.565(13)	**N10 C9**	1.313(17)	**O9 P3 O12**	110.0(12)
**P1 O4**	1.567(14)	**N10 C10A**	1.338(17)	**O10 P3 O11**	109.1(12)
**Main P1 O**	1.544	**Main N C**	1.321	**O10 P3 O12**	109.5(11)
**P2 O5**	1.551(14)	**C1A C5A**	1.428(17)	**O11 P3 O12**	111.6(11)
**P2 O6**	1.550(14)	**C1A C10A**	1.393(16)	**O13 P4 O15**	108.8(12)
**P2 O7**	1.520(13)	**C2 C3**	1.417(17)	**O13 P4 O16**	107.9(11)
**P2 O8**	1.554(15)	**C3 C4**	1.400(17)	**O13 P4 O17**	112.9(12)
**Main P2 O**	1.544	**C4 C5A**	1.430(17)	**O15 P4 O16**	106.4(11)
**P3 O9**	1.571(14)	**C5 C5A**	1.419(17)	**O15 P4 O17**	107.8(12)
**P3 O10**	1.556(14)	**C5 C6**	1.377(17)	**O16 P4 O17**	112.9(12)
**P3 O11**	1.514(13)	**C6 C6A**	1.405(18)	**O14 P5 O18**	108.9(12)
**P3 O12**	1.529(12)	**C6A C10A**	1.425(17)	**O14 P5 O19**	111.8(12)
**Main P3 O**	1.543	**C7 C6A**	1.425(17)	**O14 P5 O20**	105.1(11)
**P4 O13**	1.563(14)	**C7 C8**	1.401(17)	**O18 P5 O19**	110.1(12)
**P4 O15**	1.543(14)	**C8 C9**	1.425(17)	**O18 P5 O20**	107.8(12)
**P4 O16**	1.561(14)	**Main C C**	1.412	**O19 P5 O20**	112.8(12)
**P4 O17**	1.532(14)	**P1 O1 Al5/Fe1**	159.0(14)	**O4 Al1 O5**	111.0(10)
**Main P4 O**	1.550	**P1 O2 Al2**	150.9(16)	**O4 Al1 O8**	108.7(11)
**P5 O14**	1.527(14)	**P1 O3 Al2**	146.0(16)	**O4 Al1 O11**	111.1(10)
**P5 O18**	1.554(14)	**P1 O4 Al1**	152.3(16)	**O5 Al1 O8**	109.4(10)
**P5 O19**	1.513(13)	**P2 O5 Al1**	151.7(15)	**O5 Al1 O11**	109.7(11)
**P5 O20**	1.561(13)	**P2 O6 Al2**	152.1(16)	**O8 Al1 O11**	106.9(10)
**Main P5 O**	1.539	**P2 O7 Al5/Fe1**	138.9(13)	**O2 Al2 O3**	110.4(11)
**Al1 O4**	1.695(14)	**P2 O8 Al1**	145.5(14)	**O2 Al2 O6**	111.1(11)
**Al1 O5**	1.717(14)	**P3 O9 Al2**	156.8(15)	**O2 Al2 O9**	107.7(11)
**Al1 O8**	1.728(14)	**P3 O10 Al3**	158.1(15)	**O3 Al2 O6**	109.0(11)
**Al1 O11**	1.685(14)	**P3 O11 Al1**	141.3(13)	**O3 Al2 O9**	108.8(10)
**Main Al1 O**	1.706	**P3 O12 Al4**	123.0(12)	**O6 Al2 O9**	109.9(11)
**Al2 O2**	1.712(13)	**P4 O13 Al3**	153.2(16)	**O10 Al3 O13**	109.0(11)
**Al2 O3**	1.705(14)	**P5 O14 Al3**	147.9(17)	**O10 Al3 O14**	106.9(10)
**Al2 O6**	1.714(14)	**P4 O15 Al4**	142.0(15)	**O10 Al3 O20**	109.7(10)
**Al2 O9**	1.734(14)	**P4 O16 Al4**	154.1(15)	**O13 Al3 O14**	108.7(11)
**Main Al2 O**	1.716	**P4 O17 Al5/Fe1**	142.5(14)	**O13 Al3 O20**	108.8(11)
**Al3 O10**	1.726(14)	**P5 O18 Al4**	154.4(16)	**O14 Al3 O20**	113.7(11)
**Al3 O13**	1.736(14)	**P5 O19 Al5/Fe1**	154.0(13)	**O12 Al4 O15**	109.7(10)
**Al3 O14**	1.693(13)	**P5 O20 Al3**	146.6(15)	**O12 Al4 O16**	107.5(10)
**Al3 O20**	1.705(14)	**O1 P1 O2**	115.1(13)	**O12 Al4 O18**	111.8(11)
**Main Al3 O**	1.715	**O1 P1 O3**	109.0(11)	**O15 Al4 O16**	107.0(10)
**Al4 O12**	1.703(14)	**O1 P1 O4**	110.0(12)	**O15 Al4 O18**	110.8(11)
**Al4 O15**	1.661(14)	**O2 P1 O3**	107.0(11)	**O16 Al4 O18**	109.9(11)
**Al4 O16**	1.738(13)	**O2 P1 O4**	108.0(12)	**O1 Al5/Fe1 O7**	91.8(8)
**Al4 O18**	1.694(14)	**O3 P1 O4**	107.4(12)	**O1 Al5/Fe1 O17**	91.6(8)
**Main Al4 O**	1.699	**O5 P2 O6**	107.0(11)	**O1 Al5/Fe1 O19**	97.3(7)
**Al5/Fe1 O1**	1.888(15)	**O5 P2 O7**	114.5(13)	**O7 Al5/Fe1 O17**	174.5(10)
**Al5/Fe1 O7**	1.932(13)	**O5 P2 O8**	107.5(11)	**O7 Al5/Fe1 O19**	92.3(9)
**Al5/Fe1 O17**	1.914(13)	**O6 P2 O7**	111.6(12)	**O17 Al5/Fe1 O19**	91.6(9)
**Al5/Fe1 O19**	1.833(15)	**O6 P2 O8**	108.3(12)		
**Main Al5/Fe1 O**	1.892	**O7 P2 O8**	107.8(11)		

S11. STEM on FeAlPO (and $AlPO_4$) STA-28

The [100] and [001] projections are very similar at the resolution achieved. The images in Figure S22 appears to be down [100] based on lattice parameters and so calculations were made for [100]. The template atoms are included in the simulation but, as expected, the contrast is very low for these light elements in ADF-STEM.

The model explicitly contained all the atoms in an 8×3×5 unit cell block. Consequently calculations for partial occupancy of the Al(5) site by Fe make each site either just Fe or just Al, so that the variability of intensity at the Fe site can be assessed in the image. To further enhance this, the calculation down the [100] zone axis was made for 2×3 unit cells, rather than a single unit cell followed by replication. At each Fe (Al) site in the [100] projection there are up to 8 Fe atoms in the slab thickness (8 unit cells or 11 nm thick). Thermal Diffuse Scattering was not included because of the size of the calculation and because each iteration would normally reallocate the Fe across the sites.

In the [100] projection the Fe sites are located 0.8 Å from P sites. These two projected sites are not resolved in the images presented where the achieved information transfer/spatial resolution is 2.0 Å. Hence the additional intensity due to Fe relative to Al sites appears shifted towards the nearby P sites.

For the FeAlPO sample, Figure S22 shows an example of the raw data in S22(a) and the averaged image (a mask of small discs surrounding each diffraction spot in the FFT of the ABSF image has been applied and the inverse FFT calculated) in S22(b). Figure S22(c) is of a region after ABSF filtering but without this averaging and shows the variation in intensity of the Al5 sites when partially occupied with Fe.

Figures S23 (A and B) highlight the variation in intensity of these partially Fe-substituted Al sites expected for two occupancies (50% and 75%) and indicate the dependence on sample thickness. The thinner the sample the more pronounced the variation in intensity at the Fe sites and some positions may have no Fe present. The thicker the sample the more the simulation will match to the averaged image.

Figure S24, which compares experimental averaged and simulated images for AlPO and FeAlPO STA-28 is a similar image to that in the main paper, but expanded.

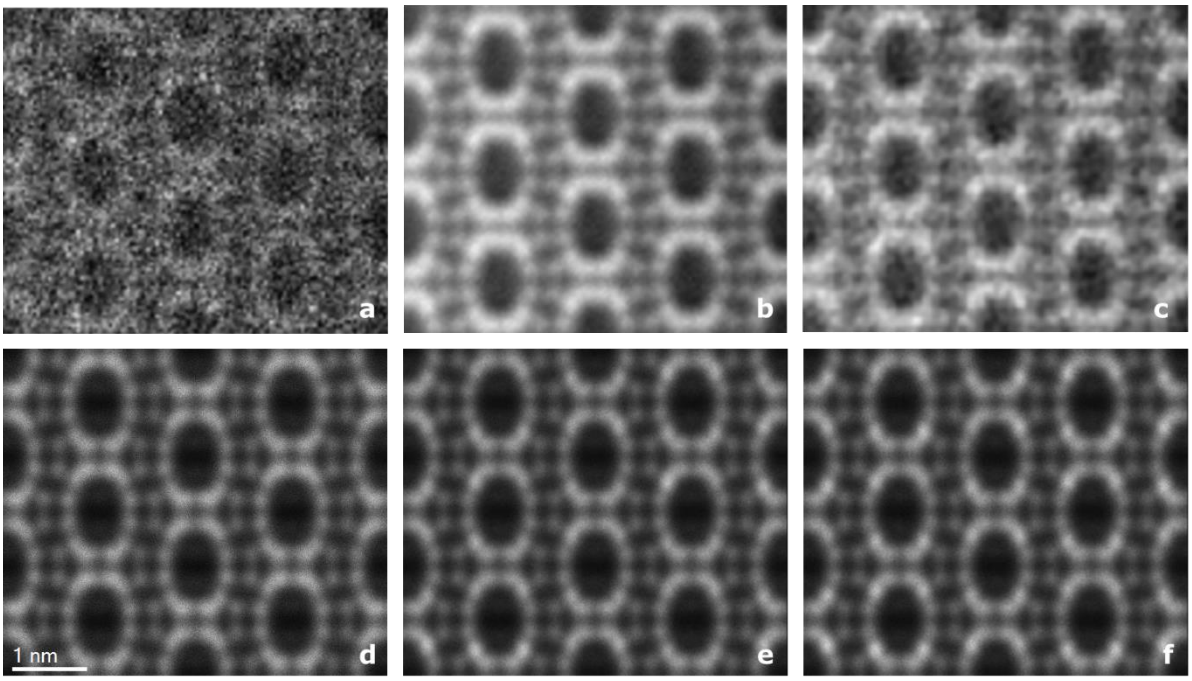

Figure S22. Experiment versus QSTEM ADF-STEM simulation of the [100] projection of the FeAlPO STA-28. The as-measured image is shown in (a), the averaged image in (b), and an area after combined ABSF and Butterworth filter is given in (c). These are compared with simulations for (d) 0% Fe, (e) 50% Fe and (f) 75% Fe on the Al5 site (11nm thick).

(A)

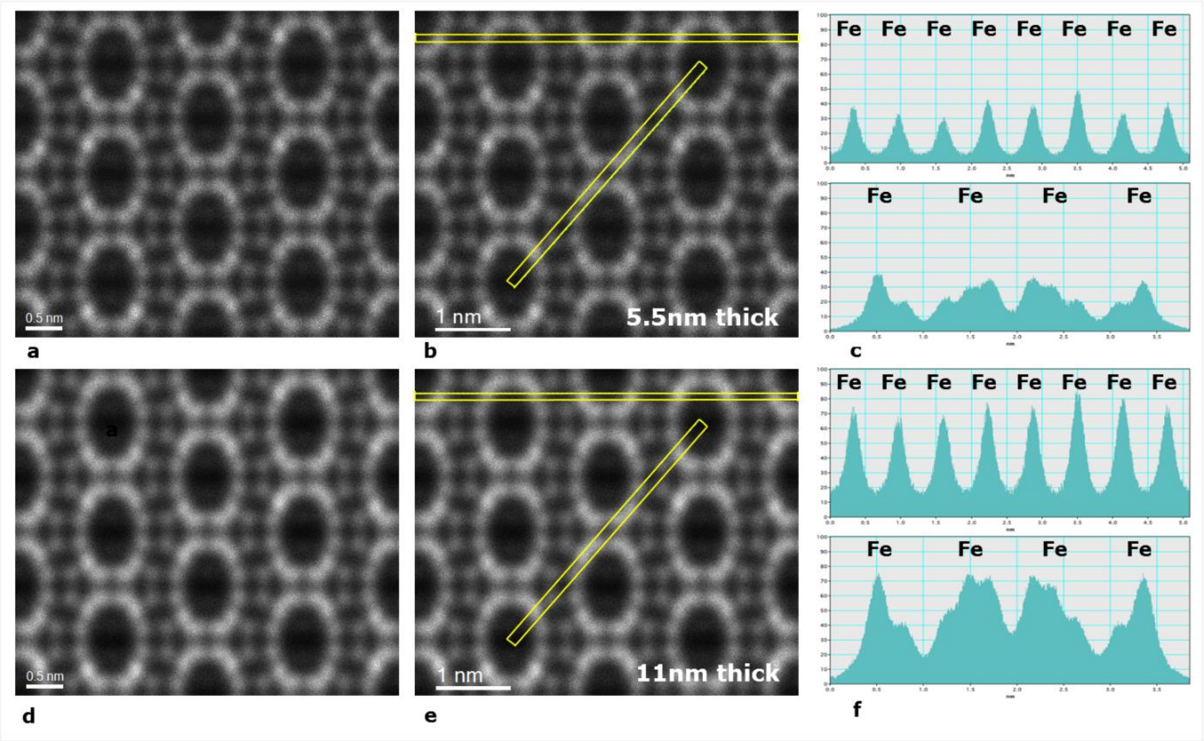

(B)

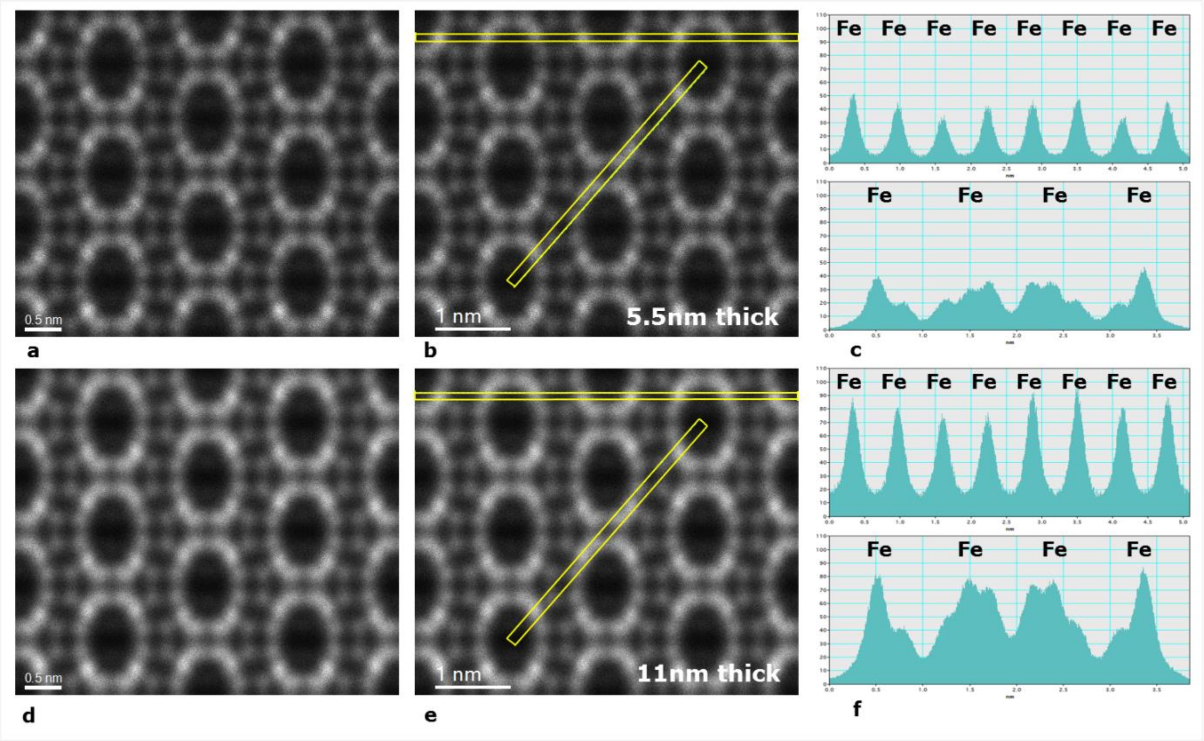

Figure S23. (A, Above) QSTEM simulations of ADF-STEM (50-200mrad) for [100] projection with 50% Fe occupancy of the Al5 site. Images (a) and (b) show the simulation for 5.5nm thick crystal whilst (c) shows profiles for the indicated lines overlaid in (b). (d) and (e) show the simulation for 11nm thick crystal whilst (f) shows profiles for the indicated lines overlaid in (e).

(B) As A above, but with 75% Fe occupancy of the Al5 site.

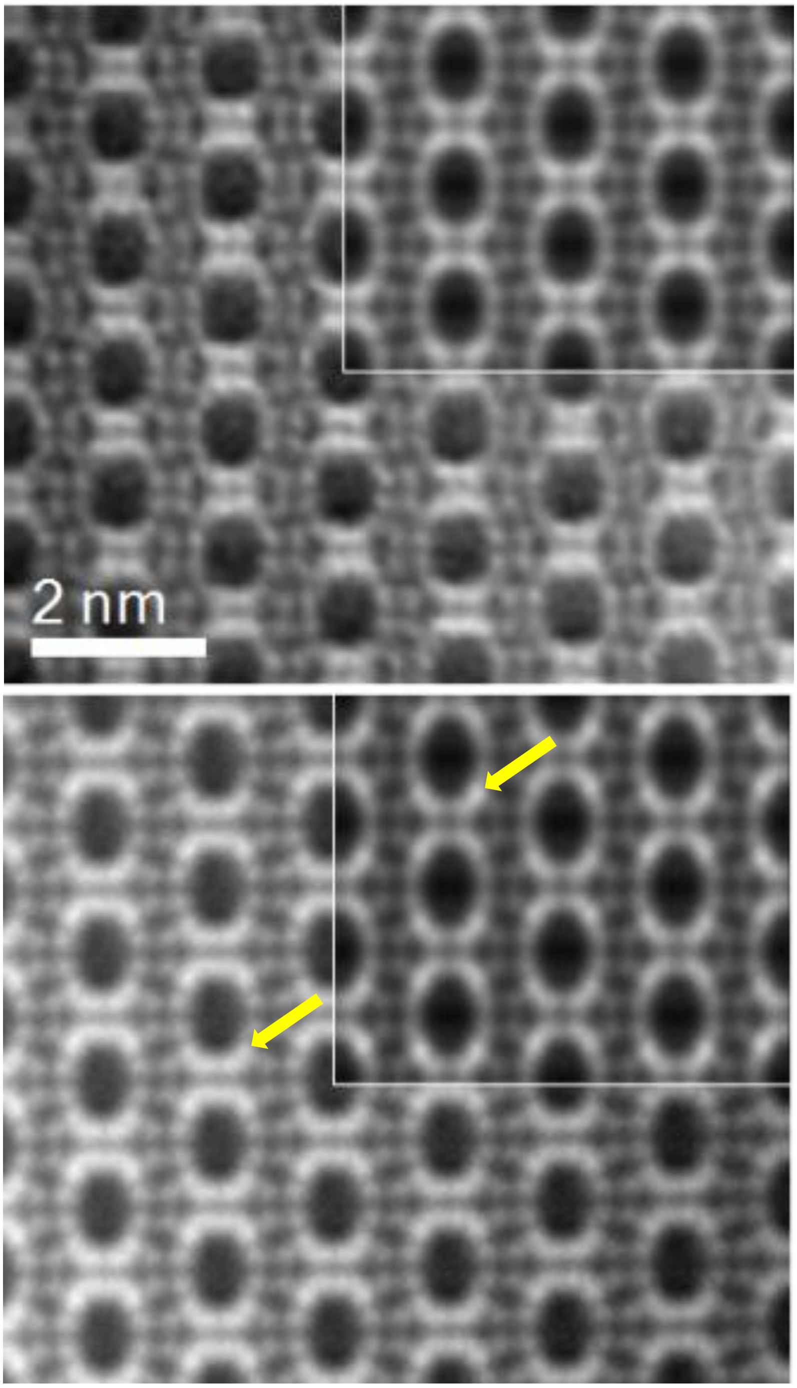

Figure 24. ADF-STEM images along [100] of as-prepared AlPO STA-28 (above) and FeAlPO STA-28 (below) processed as described above to give an averaged image. The insets are QSTEM simulations in which 0% and 75%, respectively, of ‘Al(5)’ have been replaced by Fe. Yellow arrows indicate representative positions of Fe atoms in experimental and simulated images.

S12. Characterisation of calcined STA-28

The first attempt at calcination was in flowing air in a tube furnace at 600 °C. The sample was exposed to lab air before a PXRD was measured. This gave rise to a broadened pattern (shown in Figure S25, compared with a simulated pattern using an energy-minimised structure). ^{27}Al MAS NMR (Fig. S26) gave broad resonances that suggested some structural decomposition, and the chemical shift indicated the Al was mainly tetrahedrally-coordinated.

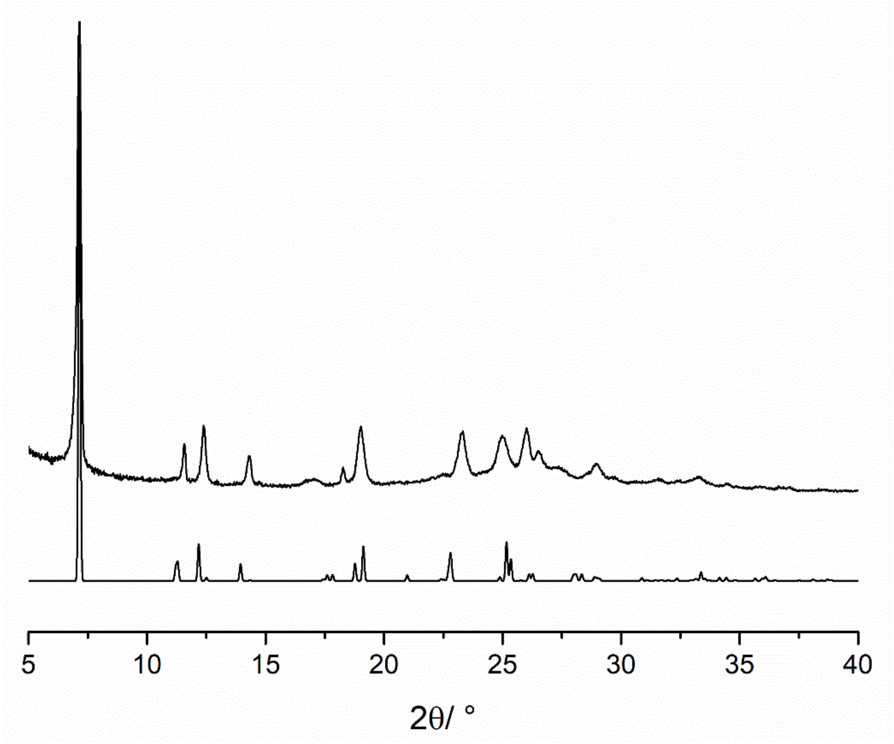

Figure S25. Powder X-ray diffraction patterns for simulated (lower) and experimental (upper) calcined AlPO STA-28 (1,10-phen).

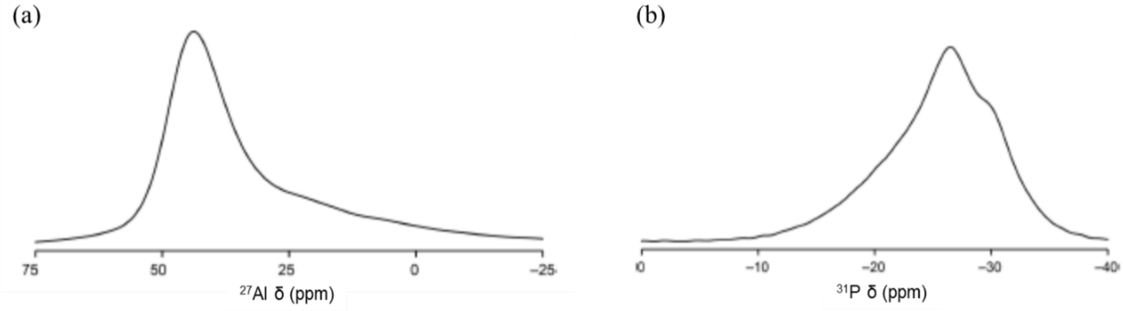

Figure S26. Solid-state NMR spectra of calcined STA-28: (a) ^{27}Al (9.4 T, 14 kHz MAS) and (b) ^{31}P (9.4 T, 14 kHz MAS).

N_2 adsorption indicated microporosity, but the isotherm was not purely Type I (Figure S27).

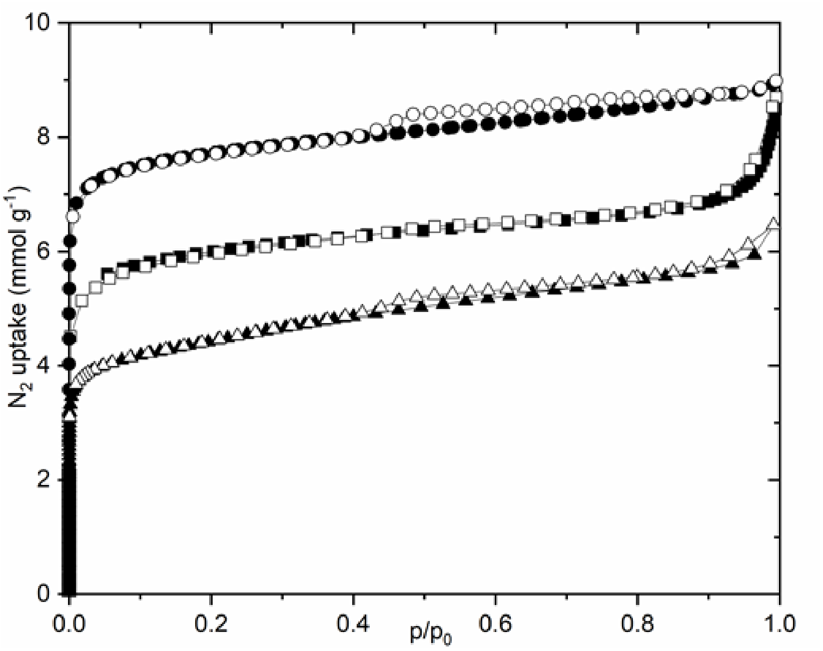

Figure S27. N_2 adsorption isotherms (at -195 °C) on calcined STA-28 samples. Triangles, AlPO STA-28 calcined in air and exposed to moist air before activation for adsorption; squares, AlPO calcined in air then stabilised by hexane adsorption on cooling in N_2 before being activated, circles, FeAlPO calcined in air at 575 °C and stabilised by hexane adsorption upon cooling in flowing N_2 before being activated. Closed symbols, adsorption branch, open symbols, desorption branch. Pore volumes were estimated by uptake at p/p_o=0.2.

S13. Energy minimised structures of AlPO and FeAlPO

AlPO STA-28

The structure of AlPO STA-28 was energy minimised using the GULP program,[S17] taking the as-prepared *I*2/*a* structure as a starting point and removing the 1,10-phenanthroline, which leaves all Al atoms four coordinated. A 2 × 2 × 2 block of the *I*2/*a* structure was allowed to relax at constant pressure to an energy minimum position without symmetry (space group *P*1) and afterwards the 'Find Symmetry' routine used to identify possible symmetry elements from the atomic coordinates. Two unit cells and symmetries were identified, which gave the same energy (to within 6.9 x 10^{-9} eV per $TO_{4/2}$ group).

*C*2/*c* *a* = 19.8806 Å, *b* = 25.3718 Å, *c* = 14.1420 Å, β =134.63°

Fddd *a* = 25.3718 Å, *b* = 20.1270 Å, *c* = 19.8806 Å

Refinements of the PXRD on calcined AlPO-28 STA-28 in both space groups were performed and gave similar fits (slightly better in *Fddd*). Refinements and structures in the *Fddd* space group are given here because of the higher symmetry and the more convenient orthogonal

cell. In the chosen *Fddd* setting there are now three Al and three P sites. The Al atom of the as-prepared structure (Al(5) of the *I*2/*a* structure) is now Al(3) in the *Fddd* cell.

The lattice energy of the fully tetrahedral STA-28 was calculated and compared with the lattice energies calculated in the same way for some known $AlPO_4$ polymorphs (GULP, constant pressure[S17]). The values obtained are consistent with those of Simperler *et al.*[S24] These values are shown graphically in Figure S28 along with the framework densities of the energy-minimised structures, and in Table S17. Figure 28 (right) plots the lattice energy per TO_2 unit, relative to berlinite (the quartz polymorph of $AlPO_4$), in the fashion of Henson and Gale.[S25] The energy per TO_2 unit was found to be ca. 4 kJ mol^{-1} less stable than frameworks with similar framework densities such as AST and CHA.

Table S17. Calculated lattice energy per unit cell, per tetrahedral cation, and also related to berlinite.

Structure	Formula	Lattice Energy / eV	Lattice Energy per TO_2 / eV	$\Delta E_{latt,berlinite}$ /eV	$\Delta E_{latt,berlinite}$ / $kJmol^{-1}$
Berlinite	$Al_3P_3O_{12}$	-804.4631	-134.0772	0.0000	0.0
AEL	$Al_{20}P_{20}O_{80}$	-5361.4619	-134.0365	0.0407	3.9
AFI	$Al_{12}P_{12}O_{48}$	-3216.5407	-134.0225	0.0547	5.3
AET	$Al_{36}P_{36}O_{144}$	-9648.9631	-134.0134	0.0638	6.2
CHA	$Al_6P_6O_{24}$	-1607.8615	-133.9885	0.0887	8.6
VFI	$Al_{18}P_{18}O_{72}$	-4822.7844	-133.9662	0.1110	10.7
AST	$Al_{20}P_{20}O_{80}$	-5358.4072	-133.9602	0.1170	11.3
LTA	$Al_{96}P_{96}O_{384}$	-25719.8280	-133.9574	0.1198	11.6
FAU	$Al_{96}P_{96}O_{384}$	-25716.7822	-133.9416	0.1356	13.1
STA-28	$Al_{40}P_{40}O_{160}$	-10713.3971	-133.9175	0.1597	15.4

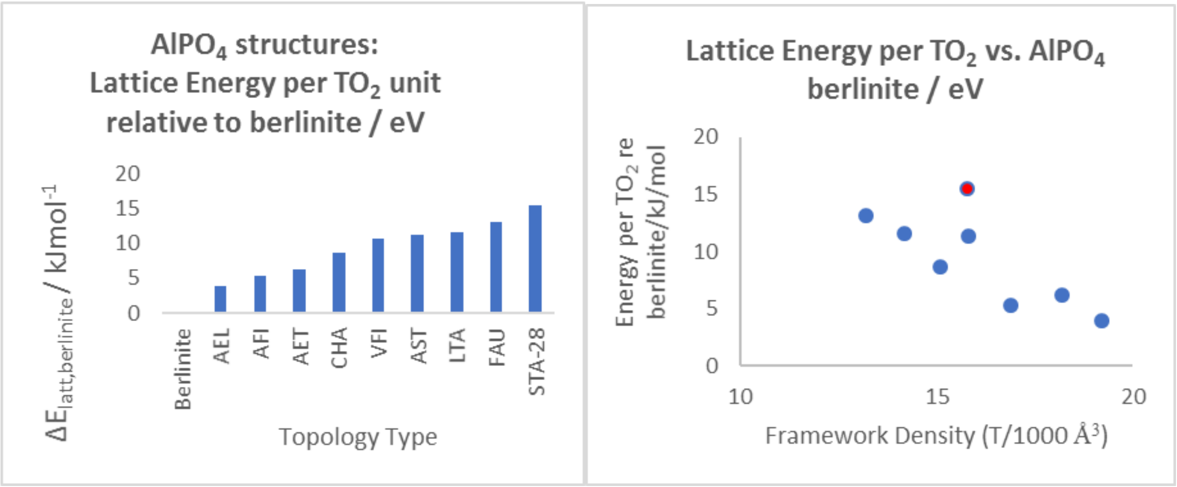

Figure S28. (Left) energy per tetrahedral cation, vs. berlinite, in $AlPO_4$ structures with different topology type, and (right) plotted as a function of framework density, with STA-28 in red.

The pore volume was calculated for the STA-28 with space group *Fddd* by generating a Connolly surface using a probe radius of 1.82 Å. For a unit cell of a = 25.37, b = 20.13, c = 19.88, the free volume was found to be 4271.22 Å^3.

FeAlPO STA-28

The optimised (template-free) structure of $FeAl_4P_5O_{20}$ STA-28, in which Fe replaces Al in every Al(5) site of the original *I*2/*a* structure was observed to adopt the *Fddd* space group, with the structural parameters given below.

Table S18. CASTEP optimised structure of $FeAl_4P_5O_{20}$ (FeAlPO STA-28). *Fddd*, *a* = 26.3633 Å, *b* = 21.2266 Å, *c* = 19.8506 Å. Optimised atomic coordinates (labels from energy minimisation in brackets).

	**Fractional Atomic Coordinates (x, y, z)**		
Fe1 (Fe15)	-0.25000	0.25000	-0.02946
Al1 (Al2)	-0.53852	0.25210	-0.63896
Al2 (Al5)	-0.16033	0.14647	-0.24853
P1(P1)	-0.15980	0.24975	-0.13690
P2 (P4)	-0.53882	0.64275	-0.24982
P3 (P16)	-0.25000	0.04179	-0.25000
O1 (O3)	-0.18844	0.25495	-0.06912
O2 (O6)	-0.16979	0.18503	-0.17040
O3 (O7)	-0.17708	0.30256	-0.18521
O4 (O8)	-0.60338	0.25718	-0.61878
O5 (O9)	-0.02946	0.18240	-0.18548
O6 (O10)	-0.59590	0.62532	-0.25522
O7 (O11)	-0.50501	0.58331	-0.24777
O8 (O12)	-0.52269	0.68145	-0.31252
O9 (O13)	-0.20167	0.08263	-0.25411
O10 (O14)	-0.24666	0.49980	-0.68647

S14. Rietveld refinement on calcined materials

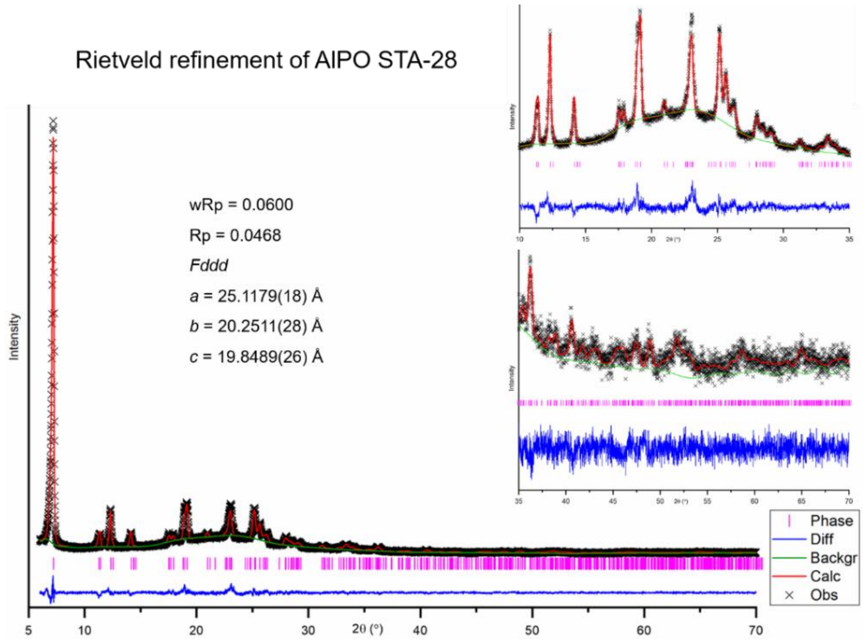

Figure S29. Rietveld plot of calcined $AlPO_4$ STA-28, after calcination to remove template, stabilisation by addition of hexane vapour when cooled in N_2 to 50 °C, followed by removal of hexane under vacuum at 50 °C.

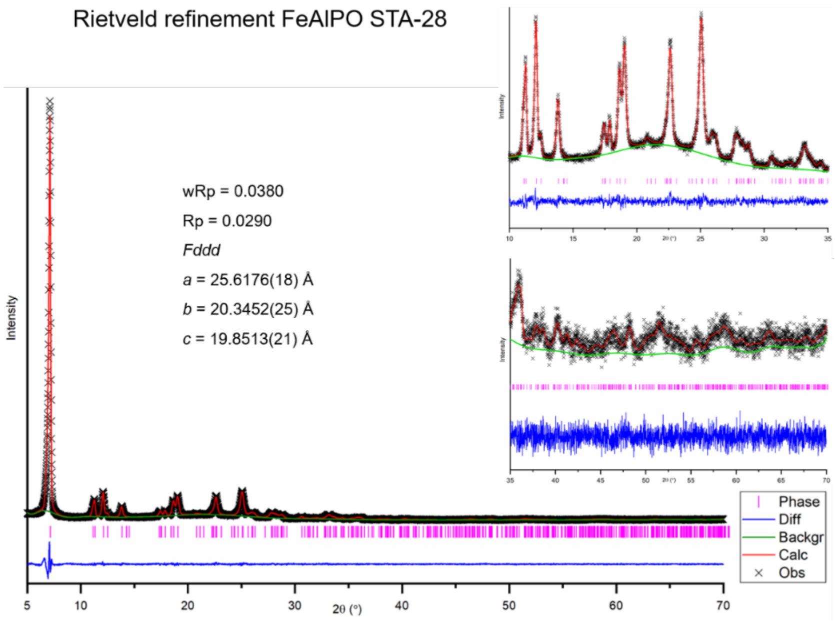

Figure S30. Rietveld plot of calcined $FeAlPO_4$ STA-28 (Fe/P = 0.155), after calcination to remove template, stabilisation by addition of hexane vapour when cooled in N_2 to 50 °C, followed by removal of hexane under vacuum at 170 °C for 8 h.

Table S19. Crystallographic details obtained from Rietveld refinement

	Calcined $AlPO_4$ STA-28	Calcined FeAlPO STA-28
**Unit cell**	$Al_5P_5O_{20}$	$Al_{4.57}Fe_{0.43}P_5O_{20}$
**Temperature/K**	298	298
**Space group**	*Fddd*	*Fddd*
**X-ray source**	Cu	Cu
**Diffractometer**	Stoe	Stoe
**Wavelength (Å)**	1.54056	1.54056
**a/ Å**	25.1175(19)	25.6157(17)
**b/ Å**	20.2478(28)	20.3444(24)
**c/ Å**	19.8465(26)	19.8524(20)
**Volume/Å^3**	10093.4(18)	10345.8(16)
**R_p**	0.0465	0.0293
**R_{wp}**	0.0596	0.0382
χ^2	1.631	1.839

Table S20. Fractional atomic coordinates, occupancies and U_{iso} parameters obtained from Rietveld refinement against PXRD data for calcined $AlPO_4$ STA-28 and calcined FeAlPO STA-28.

Calcined $AlPO_4$ STA-28	x	y	z	Occup.	Mult.	Uiso
**P1**	0.2837(4)	0.8753(6)	0.7674(5)	1.0	32	0.0213(9)
**P2**	0.1564(4)	0.7592(4)	0.8721(5)	1.0	32	0.0213(9)
**P3**	0.375	0.6635(7)	0.875	1.0	32	0.0213(9)
**Al1**	0.1603(4)	0.8717(6)	0.7599(5)	1.0	32	0.0213(9)
**Al2**	0.2843(4)	0.7720(5)	0.8736(7)	1.0	32	0.0213(9)
**Al3**	0.375	0.875	0.6546(8)	1.0	32	0.0213(9)
**O1**	0.31979(24)	0.8789(9)	0.7021(5)	1.0	32	0.0213(9)
**O2**	0.3008(5)	0.8178(8)	0.8093(8)	1.0	32	0.0213(9)
**O3**	0.2886(6)	0.9400(7)	0.8035(8)	1.0	32	0.0213(9)
**O4**	0.22619(29)	0.8641(9)	0.7422(6)	1.0	32	0.0213(9)
**O5**	0.1423(6)	0.8039(6)	0.8084(7)	1.0	32	0.0213(9)
**O6**	0.21950(28)	0.7447(6)	0.8643(9)	1.0	32	0.0213(9)
**O7**	0.1285(5)	0.69318(27)	0.8621(6)	1.0	32	0.0213(9)
**O8**	0.1513(6)	0.8090(7)	0.9374(6)	1.0	32	0.0213(9)
**O9**	0.32406(23)	0.7047(5)	0.8744(9)	1.0	32	0.0213(9)
**O10**	0.3752(5)	0.6203(6)	0.81069(27)	1.0	32	0.0213(9)
**Calcined FeAlPO STA-28**	**x**	**y**	**z**	**Occup.**	**Mult.**	**Uiso**
**P1**	0.2848(4)	0.8772(6)	0.7628(4)	1	32	0.02792
**P2**	0.1603(4)	0.7628(4)	0.8738(5)	1	32	0.02792
**P3**	0.375	0.6642(6)	0.875	1	16	0.02792
**Al1**	0.1625(4)	0.8749(6)	0.7643(4)	1	32	0.02792
**Al2**	0.2857(4)	0.7697(5)	0.8764(6)	1	32	0.02792
**Al3**	0.375	0.875	0.6557(6)	0.575(15)	16	0.02792
**Fe3**	0.375	0.875	0.6557(6)	0.425(15)	16	0.02792
**O1**	0.32053(24)	0.8715(8)	0.7004(5)	1	32	0.02792
**O2**	0.3003(6)	0.8242(7)	0.8154(7)	1	32	0.02792
**O3**	0.2956(6)	0.9463(6)	0.7959(6)	1	32	0.02792
**O4**	0.22703(29)	0.8706(10)	0.7439(6)	1	32	0.02792
**O5**	0.1455(6)	0.8085(6)	0.8128(6)	1	32	0.02792
**O6**	0.22139(27)	0.7489(6)	0.8700(9)	1	32	0.02792
**O7**	0.1339(6)	0.69348(31)	0.8622(6)	1	32	0.02792
**O8**	0.1512(6)	0.8052(6)	0.9409(6)	1	32	0.02792
**O9**	0.32490(24)	0.7046(5)	0.8694(8)	1	32	0.02792
**O10**	0.3786(5)	0.6215(6)	0.80970(29)	1	32	0.02792

Table S21. T-O, C-N/C-C bond distances (Å) and O-T-O/T-O-T angles (°) obtained from Rietveld refinement against PXRD data for calcined $AlPO_4$ STA-28 and calcined FeAlPO STA-28.

Atoms	Calcined AlPO4 STA-28	Calcined FeAlPO4 STA-28
P1 O1	1.584(9)	1.543(9)
P1 O2	1.492(10)	1.552(10)
P1 O3	1.497(10)	1.575(10)
P1 O4	1.546(10)	1.533(10)
Mean P1 O	1.529	1.551
P2 O5	1.596(10)	1.573(10)
P2 O6	1.619(10)	1.591(10)
P2 O7	1.521(9)	1.581(9)
P2 O8	1.647(9)	1.605(10)
Mean P2 O	1.596	1.588
P3 O9 (×2)	1.527(8)	1.528(8)
P3 O10 (×2)	1.548(8)	1.563(8)
Mean P3 O	1.538	1.546
Al1 O4	1.698(10)	1.705(10)
Al1 O5	1.736(10)	1.715(10)
Al1 O8	1.764(9)	1.701(10)
Al1 O10	1.657(10)	1.708(9)
Mean Al1 O	1.714	1.707
Al2 O2	1.632(10)	1.684(10)
Al2 O3	1.642(10)	1.710(11)
Al2 O6	1.730(10)	1.705(10)
Al2 O9	1.689(9)	1.669(9)
Mean Al2 O	1.673	1.692
Al3/Fe3 O1 (×2)	1.678(8)	1.656(7)
Al3/Fe3 O7 (×2)	1.620(8)	1.654(8)
Mean Al3 O	1.649	1.655
P1 O1 Al3/Fe3	158.5(8)	157.9(8)
P1 O2 Al2	143.7(11)	152.2(11)
P1 O3 Al2	145.6(13)	135.4(9)
P1 O4 Al1	146.1(9)	151.0(10)
P2 O6 Al2	148.5(9)	154.4(10)
P2 O7 Al3/Fe3	134.8(9)	137.3(9)
Al1 O8 P2	160.6(11)	149.2(10)
Al2 O9 P3	159.3(8)	158.0(9)
Al1 O10 P3	133.6(8)	136.1(9)
O1 P1 O2	109.1(9)	109.7(9)
O1 P1 O3	107.7(8)	107.4(8)
O1 P1 O4	106.0(7)	111.7(8)
O2 P1 O3	113.1(8)	107.1(8)
O2 P1 O4	109.6(9)	110.5(9)
O3 P1 O4	111.1(9)	110.3(9)
O5 P2 O6	104.1(8)	107.8(9)
O5 P2 O7	107.0(8)	108.2(8)
O5 P2 O8	105.0(7)	106.6(7)
O6 P2 O7	106.2(8)	104.8(8)
O6 P2 O8	105.2(8)	106.1(8)
O7 P2 O8	127.3(9)	122.6(9)
O9 P3 O9	113.8(11)	114.9(11)
O9 P3 O10 (×2)	107.7(5)	106.7(5)
O9 P3 O10 (×2)	108.2(5)	108.0(5)
O10 P3 O10	111.1(11)	112.5(11)
O4 Al1 O5	107.3(8)	109.9(8)
O4 Al1 O8	108.7(8)	109.3(8)
O4 Al1 O10	110.0(7)	106.7(7)
O5 Al1 O8	105.5(7)	108.8(7)
O5 Al1 O10	108.4(7)	108.8(7)
O8 Al1 O10	116.5(8)	113.4(8)
O2 Al2 O3	114.0(8)	110.4(7)
O2 Al2 O6	109.6(8)	108.9(8)
O2 Al2 O9	108.4(8)	109.2(8)
O3 Al2 O6	107.8(8)	108.2(8)

O3	Al2	O9	109.4(8)	107.9(8)
O6	Al2	O9	107.4(6)	112.2(7)
O1	Al3/Fe3	O1	111.7(10)	115.1(9)
O1	Al3/Fe3	O7 (×2)	112.2(5)	111.1(5)
O1	Al3/Fe3	O7 (×2)	102.0(5)	101.5(5)
O7	Al3/Fe3	O7	117.2(11)	117.2(10)

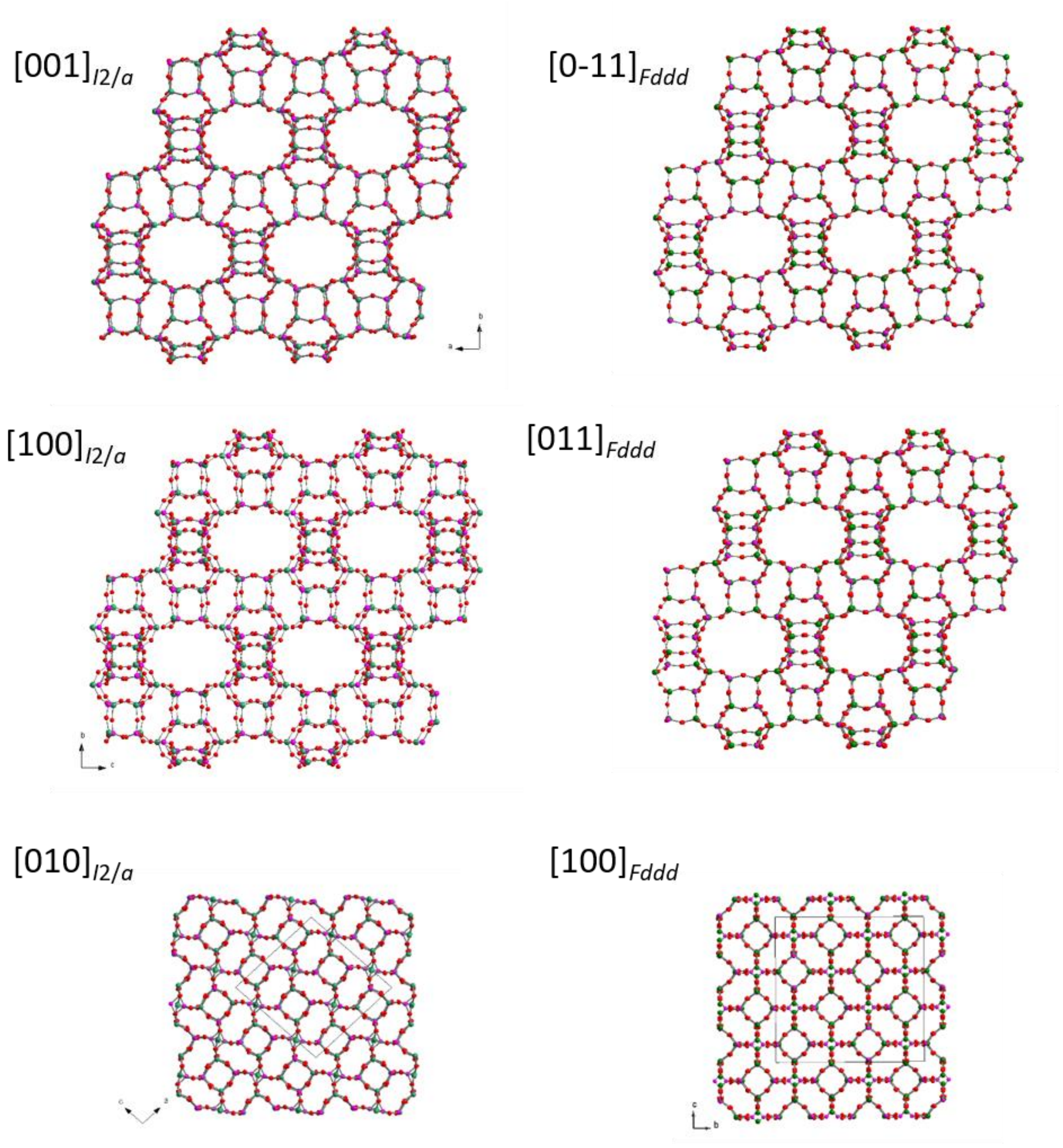

Figure S31. Comparison of framework structures of (left) as-prepared and (right) calcined $AlPO_4$ STA-28 viewed down comparable directions.

References

[S1] A. Turrina, R. Garcia, P. A. Cox, J. L. Casci, P. A. Wright, *Chem. Mater.* **2016**, *28*, 14, 4998–5012.

[S2] CrysAlisPro: Data Collection and Processing Software, Rigaku Corporation, Tokyo 196-8666, Japan, 2015.

[S3] G. M. Sheldrick, *Acta Crystallogr. A*, **2008**, *A64*, 112–122.

[S4] CrystalStructure 4.2: Crystal Structure Analysis Package, Rigaku Corporation, Tokyo 196-8666, Japan, 2000.

[S5] K. J. Pike, R. P. Malde, S. E. Ashbrook, J. McManus, S. Wimperis, *Solid State Nucl. Magn. Reson.* **2000**, *16*, 203–215.

[S6] Z. Klencsar, *Nucl. Instrum. Methods Phys. Res. Sect. B Beam Interact. Mater. At.* **1997**, *129*, 527–533.

[S7] M. Blume, J. A. Tjon, *Phys. Rev,* **1968**, *165*, 446–456.

[S8] C. T. Koch, Arizona State University. PhD thesis. **2002**

[S9] R. Kilaas, *J. Microscopy,* **1998**, *190*, 45-51

[S10] G. M. Sheldrick, *Acta Crystallogr. A*, **2014**, *A70*, C1437.

[S11] A. J. C. Wilson, Ed., *International Tables for Crystallography*, Table 6.1.1.4, Kluwer Academic Publishers, Dordrecht, Netherlands, **1992**, *vol. C*.

[S12] J. A. Ibers, W. C. Hamilton, *Acta Crystallogr.* **1964**, *17*, 781.

[S13] D. C. Creagh, W. J. McAuley, *International Tables for Crystallography* Table 4.2.6.8, Kluwer Academic Publishers, Boston, **1992**, *vol. C*.

[S14] D. C. Creagh, J. H. Hubbell, *International Tables for Crystallography*, Table 4.2.4.3, Kluwer Academic Publishers, Boston, **1992**, *vol. C*.

[S15] A. C. Larson, R. B. Von Dreele, Los Alamos Natl. Lab. Rep. LAUR.

[S16] C. J. Howard, *J. Appl. Crystallogr.* **1982**, *15*, 615–620.

[S17] Gale, J.D. *J. Chem. Soc., Faraday Trans.* **1997**, 629.

[S18] M. J. Sanders, M. Leslie, C. R. A. Catlow *J Chem Soc Chem Commun*. 1984, 1271.

[S19] Dassault Systèmes, BIOVIA, Materials Studio 2019, San Diego.

[S20] S. J. Clark, M. D. Segall, C. J. Pickard, P. J. Hasnip, M. J. Probert, K. Refson and M.C. Payne, *Z. Kristallogr.*, **2005**, *220*, 567

[S21] D. M. Dawson, V. R. Seymour, S. E. Ashbrook, *J. Phys. Chem. C*, **2017**, *121*, 28065-28076.

[S22] J. L. Jorda, L. B. McCusker, C. Baerlocher, C. M. Morais, J. Rocha, C. Fernandez, C. Borges, J. P. Lourenco, M. F. Ribeiro, Z. Gabelica, *Microporous Mesoporous Mater.* **2003**, *65*, 43-57.

[S23] J. K. Lee, J. Shin, N. H. Ahn, A. Turrina, M. B. Park, Y. Byun, S. J. Cho, P. A. Wright, S. B. Hong, *Angew. Chem. Int. Ed. Engl.* **2015**, *54*, 11097–11101.

[S24] A. Simperler, M. D. Foster, R. G. Bell, J. Klinowski, *J. Phys. Chem. B* **2004**, *108*, 869-879.

[S25] N. J. Henson, A. K. Cheetham, J. D. Gale, *Chem. Mater.* **1996**, *8*, 664-670.

Crystallographic cif files

Single crystal data	STA28-SCXRD.cif
Powder data	AlPO_FeAlPO_STA-28.cif
Energy-minimised structure simulations	STA28_fddd_AlPO-FeAPO-sim.cif
